# The development of social sustainability through traditional sporting games. A temporal approach with the Elbow Tag

**DOI:** 10.1371/journal.pone.0312092

**Published:** 2024-11-25

**Authors:** Pere Lavega-Burgués, Rafael Luchoro-Parrilla, Paula Pla-Pla, Miguel Pic

**Affiliations:** 1 Motor Action Research Group (GIAM), INDEST, Institut Nacional d’Educació Física de Catalunya (INEFC), Universitat de Lleida (UdL) Partida la Caparrella, Lleida, Spain; 2 Motor Action Research Group (GIAM), INDEST, Expert in Traditional Canarian Games and Sports of the Telde Town Hall and the Government of the Canary Islands, Spain; 3 Motor Action Research Group (GIAM), INDEST, Institut Nacional d’Educació Física de Catalunya (INEFC), Universitat de Barcelona (UB), Barcelona, Spain; 4 Motor Action Research Group (GIAM), University of Valladolid, Valladolid, Spain; Opole University of Technology: Politechnika Opolska, POLAND

## Abstract

This manuscript is based on two studies that investigate the process of social construction of time in the two versions of traditional sports game (TSG) known as Elbow Tag. The internal logic of this TSG has an original role-change system. Changing a rule in version 1 (V1) implies a major modification of the role system in V2. It is a mixed methods design employing an associative and interpretive strategy. It involved 140 participants in two eight-minute games V1 and V2. Different statistical techniques (cross-tabulations, classification trees, T-Patterns) were used to analyse data on motor behaviours (external observation), and content analysis of the strategic and emotional meaning of motor conducts (internal gaze). Multidimensional internal temporal units were used: changes of subroles, (cognitive) number of interactions (relational) and time required for different physical efforts (organic). The temporal plot is constructed by deciphering the exchanges between the Cat, Mouse and Pitcher roles. In V1 and V2 of the Elbow Tag game, each Role caused a singular and multidimensional subjective time. This fact can be observed through the strategic chains, their decisional and emotional interpretation. The findings favour an innovative physical education, in order to educate the students’ temporal motor conducts.

## 1. Introduction

### 1.1. Time, society and culture. The civilizing process of physical and social time

Since birth, individuals learn to imbue a temporal order and meaning to the succession of actions that accompany their daily lives. According to Elias [1], the time:

“Symbol of a relationship that a human group (that is, a group of living beings with the biological ability to reconcile and synthesize) establishes between two or more processes, among which it takes one, as a frame of reference or measure for the others.”

In highly urbanized and industrialized societies, humans rely heavily on instruments to measure and regulate time. Devices like clocks function as tools, enabling individuals to navigate the continuum of social and natural processes in which they are immersed. Clocks serve a crucial role in helping individuals to orient themselves and regulate their behaviour, facilitating thereby coordination with others [1].

Historically, time has been portrayed as a natural given, often referred to as ’objective time,’ presumed to exist independently of humanity. Einstein challenged this notion, asserting that time is a form of relationship known as ’social time,’ distinct from Newton’s conceptualization of it as an objective flow inherent in nature, akin to rivers or mountains. Social time represents a ’subjective’ construct rooted in human nature, signifying a social or cultural dimension. Rather than being opposing terms, they are interconnected concepts in every person’s life. Time, beyond its physical or objective aspect (physical time) tied to seconds, minutes, hours, or days separating events, also encompasses a realm of symbols and cultural meanings arising from the social actions we engage in. Each person learns to perceive and internalise time through a singular subjective experience (psychological time) in the context of his or her society [2, 3]. In addition to physical (objective) time, cultural (social) time functions as a social construct, varying across societies. The perception and conception of this cultural time shape the behaviours and relationships of community members [1].

As individuals mature, they acquire an understanding of the customary temporal cues within their society (social time), shaping their conduct accordingly (subjective experience of time or psychological time). Elias [1] formulates his theory based on the concept of the civilizing process. According to this theory, as a society undergoes increased complexity and heightened social interactions, there emerges a necessity for a more intricate regulation of individual behaviours, essential for sustaining social cohesion and preventing conflicts. This civilizing process prompts individuals to internalize societal norms, values, and restrictions (external social coercion) while also managing and tempering their impulses and behaviours (individual self-coercion). Consequently, individuals learn to align their ’physiological clock’ with the ’social clock’ and subject it to self-discipline [1, 4, 5].

### 1.2. The traditional sporting game as a civilizing agent of social time

Among the various social scenarios that facilitate the civilizing process of society Elias [1], Traditional sporting games (TSG) deserve special attention. TSG are motor practices with rules rooted in local tradition, associated with a specific way of experiencing a wide variety of interpersonal relationships and subject to different temporal structures [4] Muñoz-Arroyave et al (2021). Each TSG has an internal logic or identity card that helps players to interconnect the subjective experience of time with objective physical processes.

Game invites the person to live a singular experience, which takes place within specific temporal and spatial limits, which is felt as if it were part of everyday life [6].

According to Turner [7], TSG operates as a ritual that generates a specific cultural time, giving rise to unique temporal experiences loaded with deep emotional meaning. Games, according to this author, trigger intense experiences from which temporary communities emerge. Through regular participation in a TSG, individuals share relationships, emotions, and a symbolic time that transcends ordinary temporal boundaries. Players engage in a playful ritual [8] uniting in a temporary sense of connection and belonging, leading them to a state of absorption in the present and setting aside daily concerns. During play, individuals enter a liminal time or liminality [7] corresponding to a state of transition and temporal transformation where the space and time of action exist apart from usual social structures.

Through TSG, individuals learn that time is not merely an abstract measure related to physical and quantifiable processes with external objects. It also corresponds to a subjective experience filled with personal meanings and cultural symbols [9]. Thus, individuals who share TSG experiences gradually learn and internalize a way of understanding interpersonal relationships, the social or cultural time of those game sessions (parts or periods that make up a game), and intense emotions. TSG creates playful communities that are, at the same time, temporary communities and emotional communities as well [9, 10].

As Geertz states (analysed by [11]) TSG constitute "deep" moments in which playful experiences are encountered with emotional intensity (Deep play), containing conflicts, symbols, values, and meanings that go beyond simple common entertainment. In this framework of deep meanings, individuals can experience a state of flow [12], in which subjective time seems to dilate or accelerate. Players immerse themselves in an optimal experience lived with intensity in the present moment, making the maximum "carpe diem" a reality.

### 1.3. Traditional sporting games and the process of sportification of playful time

The social construction of time has undergone significant changes throughout history. Elias [1] argues that in premodern or less complex societies, the concept of time was more closely tied to natural cycles, such as seasonal rhythms or agricultural cycles. People held a more cyclical perception of time, where events were repeated and governed by pre-established patterns. In addition, time control rested in the hands of external factors, such as natural forces and deep-rooted cultural traditions. However, with the development of modern societies, particularly since the Industrial Revolution, industrialization, and urbanization transformed the concept of time towards a more linear and sequential perspective [1].

Traditional sporting games (TSGs) have not escaped this transformation of the social construction of time since the Industrial Revolution. One of the primary changes in TSGs has been their transformation into sports, a process known as sportification [13]. The pursuit of records, achieving the best performance, the emphasis on competition, and scoreboard control have favoured competitive games with rules prescribing a linear time, oriented towards a final objective [14]. Sport has exalted the control of physical time and a way of constructing social time linked to measurement and the imposition of a temporal rhythm associated with the sporting spectacle.

The institutionalization of time in these games has incorporated normative control units of physical (objective) time, such as ’time out’ (which does not count in the match duration), sudden death in tennis, passivity in handball, the three seconds to be in the opposing team’s zone in basketball, or time limits for serving in tennis. Additionally, sophisticated technological resources have been introduced to measure physical time precisely, discriminating down to thousandths of a second. This precision is crucial for institutionalized social time, evident in the scoreboard in motor racing or motorcycling, the photo finish of athletes reaching the finish line, or the notation of the official game clock to determine if the ball entered the basket in basketball before the last second of the play or game.

Sports, or institutionalized sporting games, revere linear time, associated with chains of game sequences that progress towards an outcome. To achieve this, the rules of sport rest on a structure of privileged playful relationships, such as duels between individuals (tennis, badminton, judo …) and collective confrontations between teams (soccer, basketball, volleyball …) [13]. This linear and sequential time is also present in many TSGs, although the competition is contextualized within the framework of a local festival, a regional championship, or a confrontation in a physical education class.

Unlike in sport, in TSGs there are also a broad repertoire of cyclic time. Parlebas [13] indicates that these games lack an established end and do not involve competition or classification of participants. The game concludes due to external factors, such as the will of the players or weather conditions. The rules of these games rely on a cooperative structure that lacks competition. Sometimes, the game involves the repetition of open sequences of cyclical motor actions (e.g., playing shovels on the beach), while other times, the game features cyclical transitions from one role to another (e.g., in chase games where the hunter and the hares swap roles or in the four corners game where the player in the centre swaps roles with a corner player).

These two groups of games, whether with or without a final score [13], open or closed [10], push players into a different notion of social or cultural time, triggering unequal meanings and social interactions [15]. In games favouring linear social time, the experience emphasizes the result and the proclamation of a winner. In contrast, games with cyclical social time prioritize the process, focusing on non-quantified exchanges in interactions between players [15].

### 1.4 Traditional sporting games at the service of social time education

Physical education can play an essential role in learning time in all its dimensions. However, teachers often focus their interventions on the education of physical time, where external units of measurement correspond to physical reality (e.g., speed to traverse a space, use of seconds, minutes or hours to calculate the duration of a task …). The education of personal time (e.g., the inner temporal experience that a sequence of actions has for each person) in the context of collective or social time (e.g., the subjective duration that each person perceives when being imprisoned or chasing an adversary in a sociomotor game) hardly receives attention [15].

In formal physical education classes and also in non-formal education contexts, the use of traditional sports games has the advantage of favouring contextualised learning situations. Performing a jump (isolated motor action) will have a different meaning, depending on whether the context corresponds to a jump in the game of hopscotch, a jump to intercept the ball in the game of dodgeball or a jump in the game of jump rope.) Each of these jumps takes in a specific physical time (objective duration, measurable with a stopwatch) and corresponds to how each pupil internalises the subjective temporal experience of jumping in the context of each game.

In addition, the TSG provides temporary learning contexts based on social norms or rules. The game acts as a miniature society, singular according to its rules [13]. Thus, by putting the rules into action, the game logically organises all interventions, i.e. it has an internal logic or singular organisational pattern. Each student, when participating in a game, tries to adapt in the best possible way to this internal logic, i.e. to the way of relating to the other participants (partners, opponents), to the space (ways of occupying the areas), to the objects (ways of using the materials) and to the time (the way of starting, passing and ending a game) [13].

Game experiences, although apparently simple, trigger deep learning, as they activate the student’s whole personality in a unitary way. Each motor action, such as throwing a ball, running after an opponent, or jumping to overcome an obstacle, mobilises the deepest part of the person; they are motor actions endowed with internal meaning, i.e., according to motor praxeology, they are motor conducts endowed with internal meaning.

In this profound experience, through motor conducts, the person transforms the external units of physical time, quantified in seconds or minutes, into subjective units of social time, referring to the internal duration it has for each person. The personal experience of the time of a game sequence (for example, passing the ball to a partner in the game of dodgeball) involves the simultaneous internalisation of the time referred to a) decision making (having decided to make the pass in a specific way); b) the motor interaction with other people (pass the ball to a teammate, to conclude a play preceded by other passes between players of the same team); c) the management of physical effort (temporal perception of the physical effort made in going from one end of the court to the other to pass the ball; d) or the expression of an emotion (celebrating the joy of receiving the ball and catching an opponent), as well as the affective perception of the duration of an episode of the game, when playing a favourable role, which is liked (e.g. when being alive, time passes very quickly) or when remaining in an unfavourable role, which is to be avoided (e.g., in the role of prisoner, time passes very slowly).

The external unit of measurement (the objective clock) is now a multidimensional subjective time unit, where cognition, relationship, emotion and physical effort are different planes of a temporal reality [16, 17].

Sometimes, game experiences give rise to learning contexts that activate a unidirectional linear social time (in games with competition or final score as in many other traditional sports games). Other times, learning situations will correspond to games that originate a cyclical and open social time (this is the case of sports games without a final score, such as games based on the transition from one role to another, for example, from alive to prisoner or from hunter to hare).

Teaching the social time construction through physical education involves helping the student to participate in an educational process to civilise the different forms of time. Through play, the educator can guide the students to discipline and moderate the impulses of their personal biological clock (individual self-action, psychogenetic) to adjust them to the social clock following the rules and codes of respectful behaviour established by society or the community (external social coercion, sociogenetic) [1].

The rules of any game establish the presence of one or several ludomotor roles referring to different set of rights and prohibitions that players must abide by. While in basketball, there is a singular role (all players are authorised to perform the same actions), in football, a distinction exists between the goalkeeper (allowed to touch the ball with their hands in their area) and the outfield player (prohibited from using their hands). Based on these defined roles, players engaged in a Traditional Sporting Game (TSG) Players participating in a TSG interpret their internal logic, i.e. the way in which they should relate to others, to the material, to space and to time.

### 1.5. Elbow Tag, a traditional sporting game to educate an original physical and social time

The Elbow Tag game has historical roots as a traditional activity for adults and individuals from high social classes during the Middle Ages in Europe. Over time, it has evolved into a game widely enjoyed by children and young people [18].

**Fig 1:**
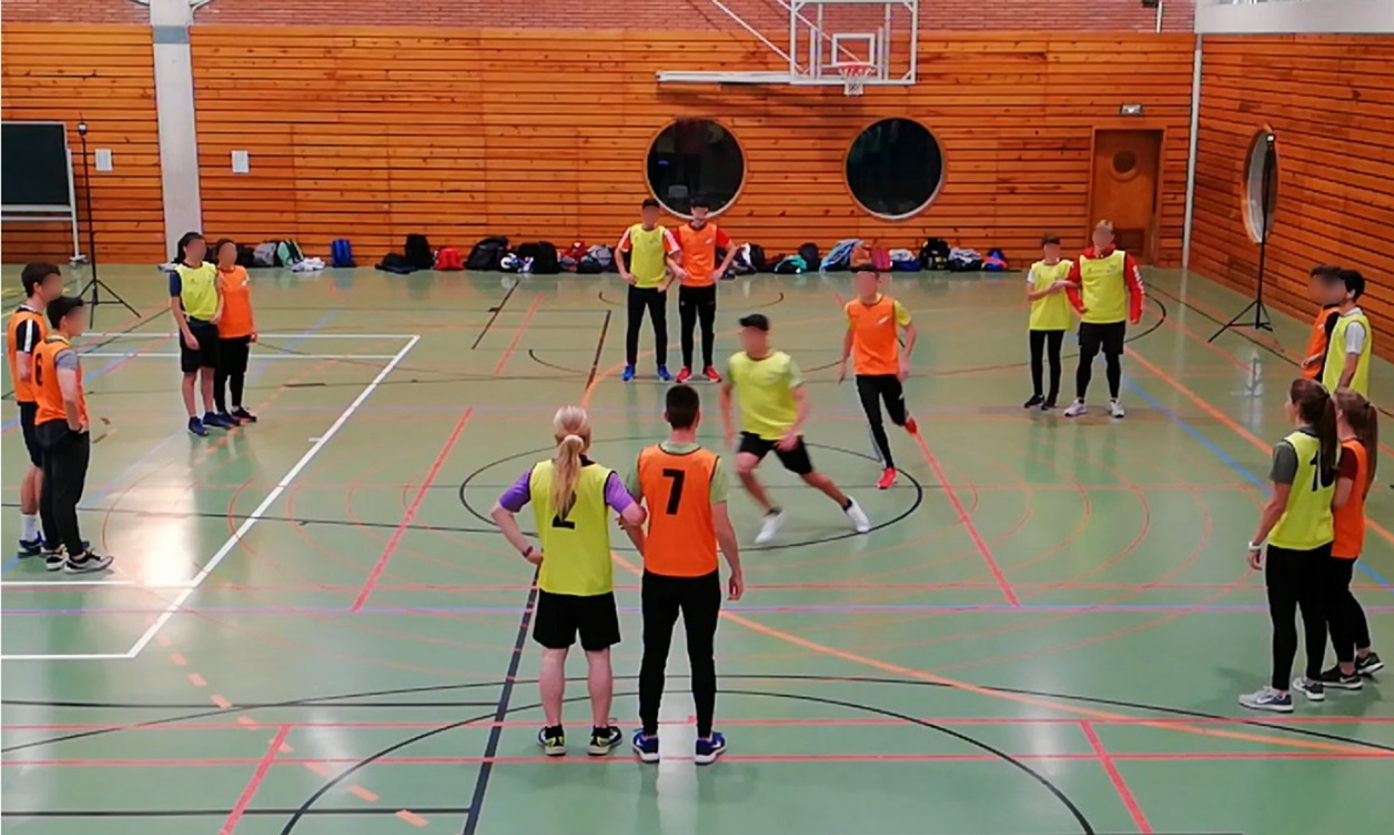
The three roles in the Elbow game. *Note*. Sixteen players can be observed practicing the Elbow tag game, with seven pairs forming a circle under the Pitcher role and a pair in the center of the image (Mouse/Cat). The player right in the middle (Mouse wearing a yellow t-shirt) is attempting to escape from player wearing an orange t-shirt (Cat role).

In this game, players assume one of three roles: Pitcher, Cat or Mouse (see Fig 1). Elbow Tag involves a unique network of motor interactions, assigning players to three roles: Pitcher, Cat, and Mouse, each leading to different decisions. In the Pitcher role, players stand in pairs, linking at elbow height to form a circle resembling a jug. These Pitcher pairs maintain a one-meter separation. Outside the circle, a player with the Cat role aims to capture another player acting as the Mouse. If the Cat successfully touches the Mouse, both players switch roles. In this scenario, the Mouse moves freely, attempting to join a Pitcher before the Cat catches it. Subsequently, the person on the opposite side of that Pitcher must leave and change roles, meaning they will take on the role of the Mouse.

This game offers two modes that influence role changes:

Version 1 (V1):
  In this version, when a player leaves the Pitcher role, they assume the role of a Mouse. Meanwhile, the previous Cat continues in their role until they successfully catch a Mouse.
Version 2 (V2):
  In this version 2 the player exiting the Pitcher, now takes on the role of Cat (G2), simultaneously switches roles with the previous Cat (G1), becoming the Mouse. This slight alteration completely transforms the network of role changes, giving rise to a gameplay sequence distinct from the previous mode (Fig 1).
  Let’s identify some of the distinctive features of the internal logic of both modalities.

#### 1.5.1. An original playful time. Original role transition

In both versions, the players are introduced into a network of interpersonal relationships arising from a constant exchange of Cat, Mouse and Pitcher roles. The rules do not establish a way of ending and proclaiming a winner (the game does not offer a unidirectional linear time). The players’ motor conducts are recurrently transiting in a network of role changes that activates cyclical time sequences. The game will end when the players, the teacher or some external cause determines it.

Motor praxeology uses graph theory to identify the system of role changes in the two versions [2]. In appearance, only one rule changes in both versions, leaving the pitcher as a Mouse (V1) or a Cat (V2). However, this minor modification will lead to major changes in the network of role changes and the consequences of the temporal intervention of the players. The following figure shows this network of role changes. White boxes signify possible role changes, while black boxes denote prohibited transitions Both versions encourage players to shift between the roles of Cat, Mouse, and Pitcher. Unlike traditional sports, the internal logic doesn’t aim to declare a winner, as it lacks a scoreboard summarizing players’ successes or failures. The temporal sequences of cyclical relationships persist until the players decide to conclude the game. Through the application of graph theory and the role change network concept of motor praxeology [2], two distinct roles change systems are identified in both modalities. Vertices represent roles, and arrowed lines indicate role changes. White boxes signify possible role changes, while black boxes denote prohibited transitions [16].

**Fig 2:**
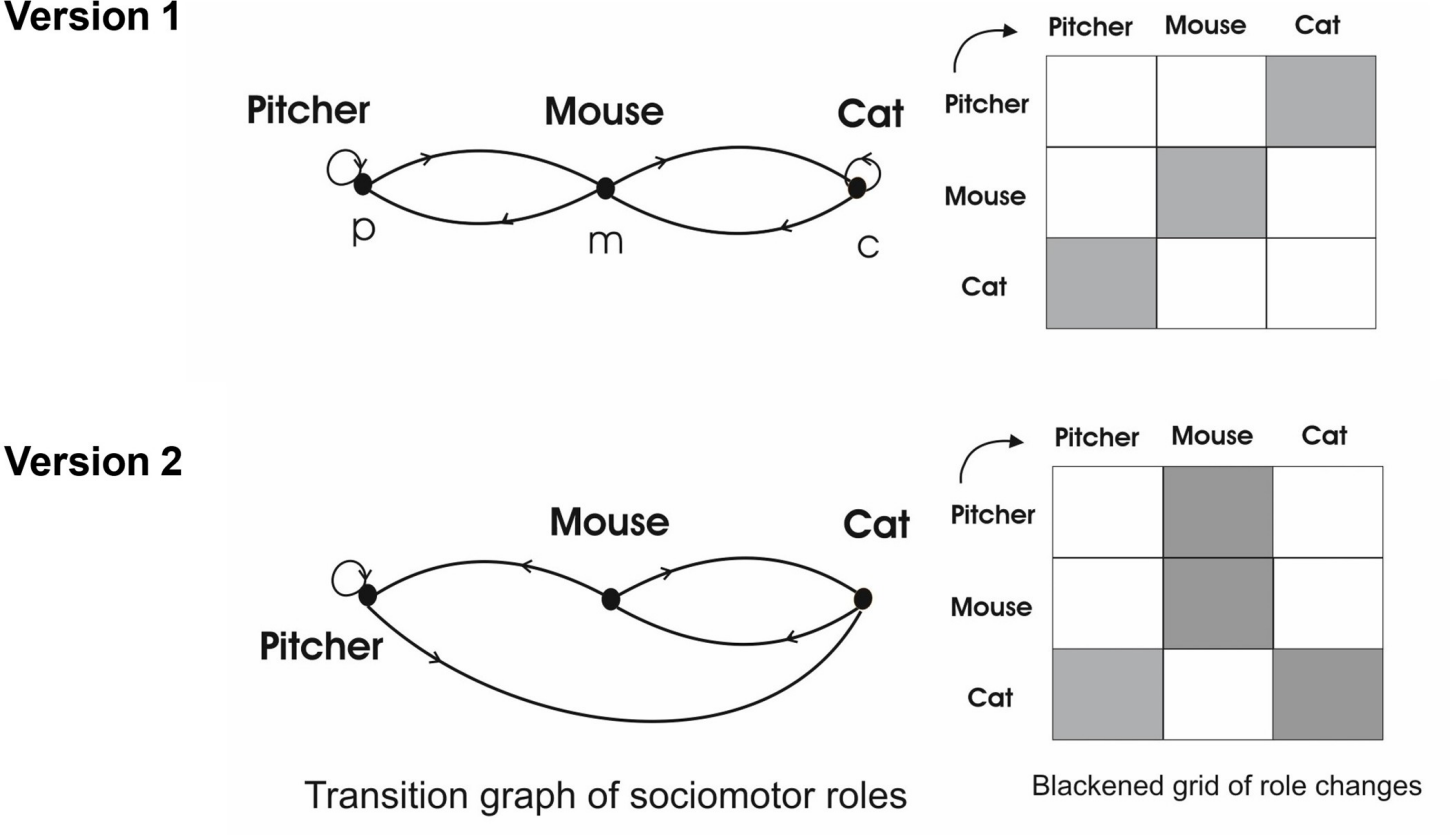
Elbow tag traditional game by roles (Pitcher, Mouse and Cat). Network and role change matrix in version 1 and 2.

In V1, the Cat can become the Mouse, and the Mouse can become the Cat, while the Pitchers continue in the same role (represented with a loop). When the Mouse changes to Pitcher, the player on the other side of the Pitcher switches to the Mouse role, while the Cat continues in the same role (loop) (Fig 2). In V2, the Cat can become the Mouse, and the Mouse can become the Cat, while the Pitchers continue in the same role (represented with a loop). The Mouse can pass to the Pitcher, causing the player on the other side of the Pitcher to switch to the Cat; this change, in turn, causes the Cat to take on the role of the Mouse.

The game, in both modalities, operates within a cyclical, recurring, and open social time. Players engage in interpreting the meaning of each other’s bodily conducts. However, the same role change, such as passing a Mouse to a Pitcher, has different consequences in each version (Fig 2). The modification in the rule of the first version introduces new interpretations, confusions, and a mix of motor interactions governed by a very brief physical time, highlighting the urgency of these relationships within the social context (subjective social time). Players must abandon the time internalised in V1 to organise their motor conduct in a different time pattern.

The motor communication network [13] in this game establishes a ternary motor interaction involving the Cat, the Mouse, and the Pitchers. This interaction revolves around the opposition between the roles of Cat and the counter-role of Mouse, where they engage in two opposing motor intentions, catch and escape. In Version 1 (V1), the Mouse’s interaction with the Pitchers introduces ambivalence. On one hand, it can lead a Pitcher to change to the role of Mouse, representing a negative motor interaction associated with a negative role change. On the other hand, the Mouse can ensure that another Pitcher person continues in their Pitcher role, signifying a positive motor interaction. Consequently, the game establishes an ambivalent network of motor interactions where players can act as both partners and opponents. The game is characterized as unstable, as these relationships of partnership or opposition can vary during the game [13, 16].

The players engaged in this game undergo unique temporal experiences characterized by the repetition of cyclic sequences, leading to a continual shift in roles—Cat, Mouse, and Pitcher. This constant rotation of roles fosters the internalization and comprehension of ambivalent interpersonal relationships, influenced by the urgency inherent in these changes of motor interactions. As a result, players experience intense emotions linked to positive or negative affective interactions, engage in intelligent decision-making driven by the interpretation of body language, and exert varied physical effort, from explosive conducts between the Cat and the Mouse to more serene conducts in the role of Elbow Tag.

The shared original playtime, rooted in a network of cyclical relational exchanges, immerses players in a state of affective transition inscribed within a liminal time [13]. This experience is rich in symbols that give rise to profound emotional meanings [7, 11]. Each game becomes a source of profound temporal sensations, fostering a unique civilizing process that contributes to a sense of belonging within the gaming community.

Understanding the temporal plot of the game requires recognizing the diverse levels players engage with as they transition through the three roles: organic (energetic), affective (emotional), cognitive (change of sub-roles or minimal decision units in each role), and relational (motor interactions). Unveiling the temporal intervals linked to cyclical social time is crucial. Role changes prompt adjustments in motor conducts, intertwined with multimodal strategic chains involving organic, cognitive, relational, and decisional elements.

At the end of the game, the players have played the same minutes (calculated with external elements of physical time, e.g., 8 minutes). However, each person has experienced a singular subjective time, depending on the temporal sequences or chains of actions they have played and, for example, the number of people they have interacted with (for example, in V1 before changing roles, a Cat can chase one, two, three or more Mice). The game is a sequence of time sequences.

To unravel the temporal plot of the game, an essential step is scrutinizing both physical time and cyclical social time within motor behaviours. External observation facilitated by tools like accelerometers or observational instruments enables the examination of players’ motor actions. Within each of the three roles, motor interventions operate cohesively, impacting the organic plane with energetic and physical effort, the cognitive plane through decision-making or sub-roles, and the relational plane involving motor interactions.

At a deeper level, investigating the temporal plot of the game delves into elucidating the significance of the playful experience for players. This entails interpreting their motor conduct, unveiling the meaning embedded in their decision-making strategy (cognitive dimension), understanding their emotional experience (experienced emotional states), exploring how they navigate relationships (interactions with others), and discerning the impact of physical effort regulation (organic dimension) on players’ internal sensations. In this context, employing a questionnaire or personalized interview is imperative.

“Within the dynamic exchange of interpersonal signs, a group microculture emerges, giving rise to the development and transmission of a cohesive system of shared norms, values, and practices. This process establishes a robust network of shared sociomotor meanings that encompasses cognitive, relational, and affective dimensions” [19].

Both external (motor behaviours) and internal (motor conducts) perspectives are pivotal in unravelling the temporal dynamics that drive the internal logic of the Elbow Tag game across its two versions. These views contribute to unveiling the social temporal plot inherent in the game.

In an increasingly globalised and diverse world, education for social sustainability has become a fundamental priority for 21st-century society. Elbow Tag, a seemingly simple traditional game, provides a valuable opportunity to foster crucial skills such as dialogue, social cohesion, and the development of interpersonal relationships. This game involves not only the physical participation of players but also facilitates the internalisation of a system of shared norms and values, promoting the creation of a "group microculture" in which cohesive sociomotor meanings are developed and transmitted. Through these dynamics, participants engage not only on a physical level but also experience a unique subjective time, shaped by their actions, decisions, and interactions with other players.

Elbow Tag acts as a setting where players internalise a singular temporal framework, structured by the internal logic of the game, which regulates their motor behaviours and conducts. This temporal framework affects not only the physical and energetic dimensions of the players but also their cognitive dimension through decision-making and their relational dimension through motor interactions. Furthermore, the emotional experience of the players and how they regulate their physical effort reflect a profound significance in their motor conduct, revealing how they navigate the social dynamics of the game.

In the context of modern education, these aspects are essential for teaching students to understand and value the importance of social cohesion and teamwork—key elements for a sustainable and equitable society. Integrating Elbow Tag into educational programmes allows students not only to participate physically in activities but also to develop skills for social living, learning to communicate, and strengthening interpersonal relationships through original motor experiences derived from traditional games.

The systemic view of the contribution of traditional sports games must recognise that they are resources to be contextualised within other everyday social activities that people engage in within their families and communities [20]. Furthermore, the future of traditional sports games should involve exploring how they can meet new social and economic demands, where enjoyment, without necessarily competing for a medal, can occur within a dynamic and evolving sports ecosystem [21].

Considering the theoretical framework of reference, two studies were conducted with the following objectives.

*Study 1*. *Referring to the social time plot of motor behaviours*.

1 Cognitive dimension. Discern the cyclical time (intervals between roles) of the players’ motor behaviour based on the network of role changes (transition time when changing roles) in the V1 and V2.
2 Relational dimension. Examine the number of relationships influencing the players’ motor behaviour by roles in Elbow Tag game (V1 and V2).
3 Organic dimension. Unveil the physical effort (magnitude vector) and steps involved in participating by roles (Cat and Mouse) in Elbow Tag game (V1 and V2).
4 Multidimensional cyclical time (360º). Reveal the temporal variables (organic, decisional and relational) of the players’ motor behaviours, with greater predictive force of the number of relationships between players activated by the V1 and V2 versions of the Elbow Tag game.
5 Multidimensional cyclical social time (360º). Identify the T-Patterns corresponding to multidimensional strategic chains (360) (relational, decisional and organic) of the players’ motor behaviours while they (players) are playing in V1 and V2.

*Study 2*. *Referring to the social time plot of motor conducts*.

6 Cognitive dimension and multidimensional meaning. To reveal the temporal plot (temporal decisional strategies) of the players’ motor conducts when participating in the three Roles in the Elbow Tag game (V1 and V2).
7 Emotional dimension and multidimensional meaning. To examine the emotional states of the motor conducts expressed by the players in the three Roles in the Elbow Tag game (V1 and V2).

## 2. Materials and methods

### 2.1. Design

This research combines two studies, utilizing mixed methods aimed to explore the game’s temporal plot. Study 1 focuses on the temporal plot of motor behaviours (quantitative data) and study 2 delves into motor conducts (qualitative data). The integration of both quantitative and qualitative data ensures a comprehensive understanding of the subject, fostering a more nuanced analysis [22, 23].

Both studies followed an associative strategy (exploring the functional relationship between variables) and corresponded to a comparative predictive design based on group comparison [24].

The use of observational methodology in Study 1 indeed constitutes a mixed methods approach [25]. The study adopted a Type III design influenced by the N/P/M observational methodology [26]. The Nomothetic (N) aspect was justified by the interest in exploring collective units of Elbow Tag game. Categorized as Punctual (P), the study involved a single recording with a specific follow-up. Aligning Parlebas’ conceptualization of roles and sub-roles [27] with criteria and categories enabled characterization of the study as Multidimensional (M).

Study 2 is a qualitative, descriptive, and interpretive investigation conducted in natural settings [28–30]. It adopts a naturalistic approach, as the study unfolds under typical conditions where university students commonly engage in their practical sessions. Simultaneously, hermeneutical units were organized in a database for statistical analysis (quantifying qualitative data) employing an associative strategy (examining the functional relationships between variables) [24]. In this instance, the statistical correlation between distinct hermeneutic units (temporal strategies in the roles of Cat, Mouse for V1 and V2) and the emotions encountered in the three roles (emotional triad in V1 and V2) was examined.

Indeed, both studies align with an interpretive investigation. The outcomes derived from both content analysis and quantitative analysis were intricately interpreted, reflecting a mixed methods approach. This interpretation is in harmony with the theoretical framework of reference, specifically rooted in the foundations of motor praxeology. In this way, the aim is to interpret the reality that the players construct of time as a mental and cognitive construction. The objective data from the system’s theoretical foundation (by analysing the internal logic and the role-change system of the two versions) are joined to the objective data of the player coming from the external observation and its statistical treatment. This objective information is merged with the subjective data from the participants’ testimonies. The analysis of internal time necessarily passes through the filter of the subjectivity of the participants. Therefore, the interpretation of the researchers of this study (observers) of the meaning conferred by the actor to their time plot is legitimised [27].

### 2.2. Participants

This study was carried out with 140 university students (*M* = 19.6, *SD* = 2.3): 43 girls (31.97%) and 96 boys (68.02%) from the first course of physical activity sciences and the sport from the University of Lleida, enrolled in the subject Theory and Practice of Motor Games. Most of the students (87%, *n* = 122) participated in some form of sport. Additionally, all students had a similar level of motor skills, as they were all deemed fit in the physical entrance tests for the studies in Physical Activity and Sport Sciences. The distribution between women and men maintained the same proportion as in the class group, with the male gender predominating over the female. The recruitment period was March of 2020.

All participants signed an express authorization allowing their filming (Declaration of Helsinki). In addition, the study was reviewed and approved by the Clinical Research Ethics Committee of the Catalan Sports Council [07/2019/CEICEGG].

The 140 participants in this study carried out the intervention divided into five groups of 25 to 35 people, according to the usual organization of the practical sessions of the subject. In each group, the participants were distributed in one or two zones (groups of up to 20 students in 1 zone; groups of more than 20 players in two zones). When intervening in two zones, one group played the Elbow Tag game version 1, and the other group played the Elbow Tag game version 2, to avoid the carryover effect on the results. The research followed the indications presented by Parlebas [16], when describing how to play Elbow Tag game.

**Fig 3:**
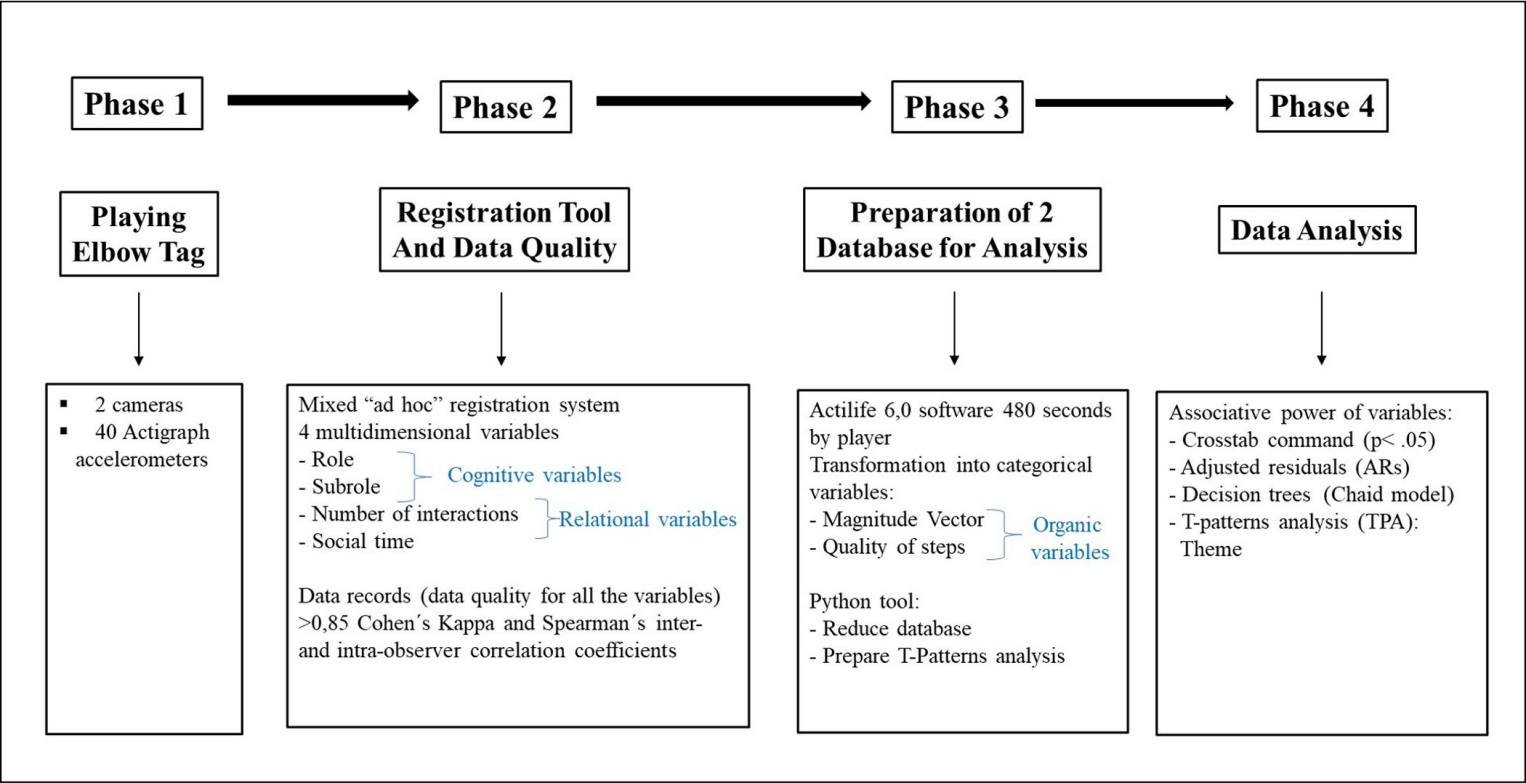
The four phases followed in the methodological strategies of this research.
Study 2. Temporal study of motor conducts.
  In the second study, the qualitative data provided by 140 participants (GES-II; V1 = 140, V2 = 140) was taken into account.

Study 1. Temporal study of motor behaviours
  In the first study, quantitative data from 15 participants (V1 = 15; V2 = 15) were examined (Fig 3). These students were randomly selected, ensuring representativeness within the conditions of the research.

### 2.3. Procedure and materials

#### 2.3.1. Study 1. Methodological strategies to analyse quantitative data on motor behaviours

*Phase 1*. *Playing Elbow Tag*. The players participated in two games, corresponding to version 1 and 2 of this game. Each game lasted 8 minutes, since this is the time used by the motor action research group (GIAM) in previous studies on other traditional sporting games.

*Phase 2*: *Registration tool and data quality*: *Cognitive dimension*. The registration of subroles corresponding to each role involved the implementation of a specialized mixed registration system within the research group (GIAM). This ad hoc system was meticulously designed, featuring exhaustive and mutually exclusive categories. It’s crucial to note that each recorded entry was specifically tied to a singular role or criterion and a corresponding sub-role or category [31]. The identification of role and sub-role categories commenced deductively, aligning with the established theoretical framework [13, 16]. Subsequently, an exploratory and inductive approach was employed, influenced by observations made by the team of observers, such as the incorporation of roles and subroles in conflict scenarios. Following the identification of all categories for the variables under observation, a comprehensive manual was crafted for the observers. This manual detailed the categories and degrees of freedom, providing a standardized guide for the documentation process.

Ensuring the precision and reliability of observation data was a priority in this study. To achieve this, a rigorous selection process was employed, enlisting four researchers who met specific criteria. These criteria included being active members of the GIAM group and possessing a minimum of two years of hands-on experience in utilizing systematic observation methodology.

*Relational dimension*. Observation instrument number of motor interactions. A continuous recording of the subroles and the number of motor interactions of each player was carried out during the 8 minutes in versions 1 and 2, following the guidelines described in another study [18].

- Observation cognitive dimension: Subroles: Pitcher: waiting POH; leaving PL; Cat: chaser CT, CC grabber; Mouse: run away MX, Mouse: catcher MC, Mouse: captured MJ; CF interpersonal conflict.
- Observation relational dimension: Range of GR relationships: number of Mice chased by the Cat: 1 (Low L); 2 (Slight SR); 3–4 (Moderate, MR); 5 or more (Hight HR).

Data analysis was performed using the SPSS software program Spss v. 25 (SPSS Inc., Chicago, IL, USA) to verify the quality of the records obtained, The Cohen’s Kappa and Spearman correlation coefficients interobserver and intra observer were applied. Consequently, 10% of the total records were selected. In pairs, while observers 1 and 2 made the first recordings, observers 3 and 4 agreed on their own recordings in separate locations. Two weeks after the end of this first phase, and respecting the same pairs of observers, the experience was repeated to record the videos again. Thus, both the intraobserver coefficients (Evaluator 1 and 2) with (Evaluator 1 and 2) or (Evaluator 3 and 4) with (Evaluator 3 and 4) and the interobserver coefficients (Evaluator 1 and 2) with (Evaluator 3 and 4) They exceeded the reliability coefficients of 0.85 accepted in social sciences (values of 0.85). This result guaranteed the quality of the records.

*Organic dimension*. Accelerometers.

To identify the exact start and end on the accelerometers, players were asked to perform several vertical jumps (for 10 s) and then remain still (for 5 s). The time of each game was previously stipulated for all groups at 8 min.

To carry out the recording procedure, two cameras (Sony DCR-SX21 model) were used. The recording time was continuous, without interruptions. 14 ActiGraph GT3X+ accelerometers (ActiGraph LLC, Pensacola, FL, USA) were used to quantify: (i) the amount of energy expended by each player, and (ii) the time spent at each of the different intensity levels. or energy while the game was going on.

- Vector magnitude of physical effort (*Vm*^*2*^): Sedentary: 0–2 CPS (S); Light (<2-34CPS) (L); Moderate >34–100 CPS (M); Vigorous: > 100 CPS (V).
- Number of steps: Low: 0–1 (L); Slight: 2 (SS); Moderate: 3 (MS); High: 4 (HS).

*Phase 3*: *Preparation of two databases for analysis*. Events were associated by seconds between each player’s set of variables. The observation procedure carried out by two observers allowed the recording of data on the role/sub-role of the variable, steps/physical effort, gender/gender interactions/number of interactions from the videos, and events developed by the players.

Data recorded by the accelerometers were downloaded using ActiLife 6.0 software (ActiGraph, Pensacola, FL, USA). The data were consolidated in periods of 1 second intervals, obtaining 480 seconds per participant. The intensity of the effort expressed by the vector of magnitudes in counts per second was transformed into a categorical variable using the cut-off points (sedentary: 0–2 CPS, light: >2 to 34 CPS, moderate: >34 to 100 CPS, or vigorous: >34 to 100 CPS). The number of steps was also considered an energy variable and was transformed into a categorical variable (low: 0–1 steps, light: 2 steps, moderate: 3 steps, or high: >4 steps).

After categorizing all variables, various statistical analyses were conducted, including cross-tabulation, decision trees, and T-patterns. Specifically, for T-pattern analysis, a cleaning process of repeated records was imperative [32]. To achieve this, a Python script was crafted using a designated tool. The objective was to eliminate instances of events recurring for more than one second consecutively (referenced in Fig 3). As an illustration, from a prior database [7], four columns were employed to represent four variables: Time Scale, 6 Categories (7 Events), Duration (Interval), and 6 Categories (2 Events). The first column documented the time in seconds for each sequence. Column 2 displayed two different sets of variables, repeated 2 times (depicted by a black circle) and 5 times (depicted by a blue circle) respectively. The third column captured the initial times of 10 and 12 seconds, along with intervals of 2 and 5 seconds, post-elimination of repeated sequences. Finally, in the fourth column, aligned with the previous one, the sequences ready for analysis were presented. In this database, six variables were analysed, interconnected through a second-by-second temporal distribution (referenced in column 1).

**Fig 4:**
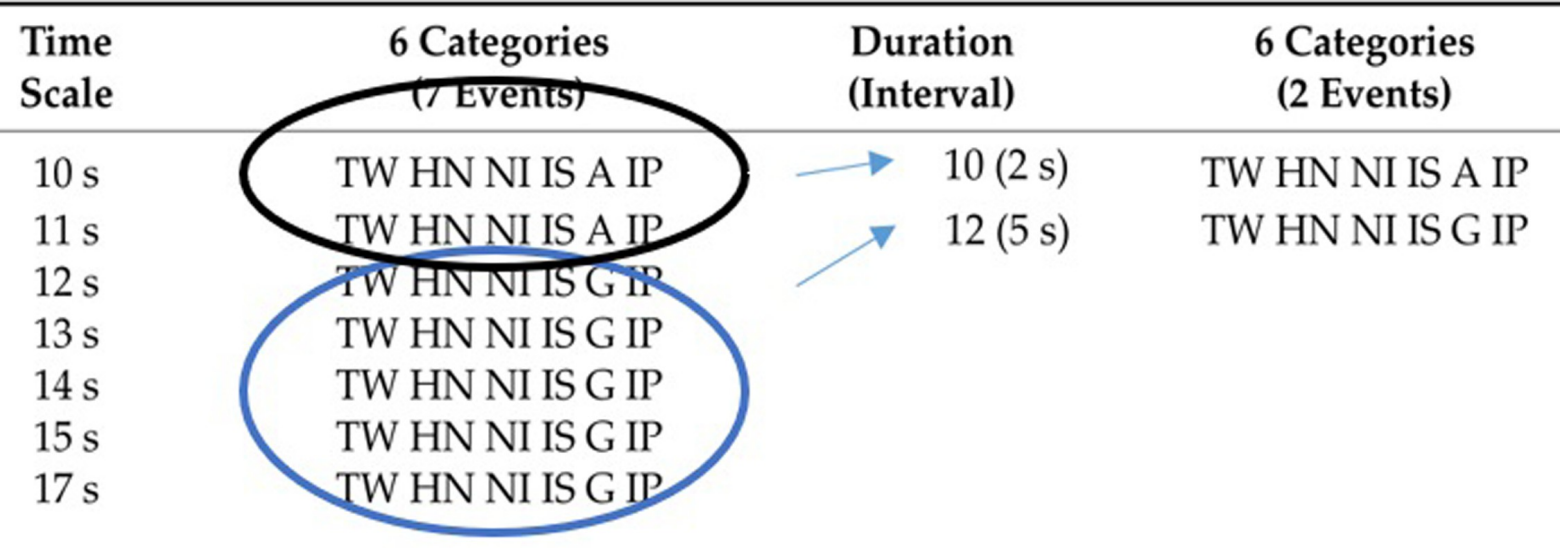
Procedure to selecting the temporal intervals associated with 2 different events. This procedure enables T-Patterns analysis.

The procedure involved eliminating rows with repeated sequences. In the second column, verification was performed, confirming the identical nature of the first (TW HN NI IS A IP) and the second event, extending to the end with repetitions of the third (TW HN NI IS G IP) and beyond. Each repetition of a set of variables (Events) extended the event intervals by at least one unit of a second. Notably, the first and third events/sequences in column 2 were included in the fourth column as they appeared for the first time. This approach facilitated the determination of event intervals, capturing the complete duration from the first appearance of an event to the next. When any of the 6 variables within the events changed state, it generated a new event, which found a place in the fourth column. The resulting database from columns 3 and 4 allowed the revelation of time intervals in players’ participation, considering not only roles but also the other variables (Fig 4).

The process successfully achieved two key objectives: firstly, it effectively reduced the size of the database, and secondly, it prepared the data for analysis using T-pattern analysis (TPA) [29].

*Phase 4*: *Statistical analysis of quantitative data*. The analysis involved assessing the associative power of variables using the Crosstab command (*p* < .05) [33] incorporating adjusted residuals (AR) >1.96 or <-1.96. Additionally, effect sizes were considered, evaluated through the Cramer’s V test, with interpretation thresholds: 0.10 = small effect, 0.30 = medium effect, and 0.50 = large effect [34].

In parallel, a supervised learning model, specifically the Chaid model, was employed for decision tree analysis. The tree construction parameters included: (i) Pearson’s chi-square test, (ii) a minimum of 100 cases for parent nodes (father *n* ≥ 100) and 50 cases for child nodes (children *n* ≥ 50), and (iii) cross-validation. These analyses were conducted using SPSS v.25 tool by SPSS Inc., based in Chicago, IL, USA.

Finally, T-Patterns Analysis (TPA) was conducted utilizing the THEME v.6 tool by Pattern Vision Ltd. based in Reykjavik, Iceland. The tool, accessible online at www.patternvision.com (accessed June 15, 2023) [32, 35] implements an algorithm to uncover sets of behaviour emerging at specified time intervals, surpassing the influence of chance. TPA identifies T-patterns, which are combinations of events occurring with a consistent temporal gap, challenging the null hypothesis that each event or behaviour is independent and randomly distributed over time. Building upon the principles outlined in [36] if A represents an earlier component and B a subsequent component of the same recurring pattern T, an interval [t + d1, t + d2] (where d2 ≥ d1 ≥ d0) is identified after an occurrence of A at t. This interval tends to contain at least one occurrence of B more frequently than expected by chance. In order to obtain the most relevant T-Patterns, we decided to eliminate the sequences of the waiting pitcher. The search focused on detecting the most complex T patterns (*p* < .05).

#### 2.3.2. Study 2. Temporal study of motor conducts

*Phase 1*. *Registration tool*. Following each game session comprising 8 minutes of play, players completed the Games and Emotion Scale (GES-II) questionnaire. Each participant individually responded to the validated GES-II sports games and emotions questionnaire [37]. His questionnaire gauged the intensity, rated on a Likert scale from 1 to 7, of five basic emotions: joy, anger, fear, sadness, and rejection.

*Temporal strategy self-perception questionnaire*. Additionally, participants provided self-perception on their temporal strategies for the roles of Cat and Mouse through a Temporal Strategy Self-Perception Questionnaire. Aligned with the principles of motor praxeology, this questionnaire sought to capture each participant’s primary temporal approach to the distinct roles within the game.

*Phase 2*. *Content analysis of qualitative data on motor conducts*. The qualitative data collected with the temporal strategy questionnaire were analysed using the "content analysis" technique with the objective of formulating valid and applicable inferences according to the context of the study [28]. The analysis followed the guidelines of previous studies (e.g., [38]) and categories were obtained using the method developed by Miles and Huberman [29], which consists of three phases: (1) reduce, (2) separate and (3) synthesize and group units of socio-emotional meaning.

*Reduction*. The initial step involved deductively reducing the transcribed comments into meaningful units, aligning with the theoretical framework.

*Separation*. Subsequently, an inductive procedure was applied to separate units of meaning, facilitating the synthesis for each role and version.

*Synthesize and group*. In the final phase, the different meaning units for each role were synthesized and grouped to facilitate a comprehensive understanding.

*Methodological integrity*. The research methodology adhered to Levitt’s guidelines on the Reporting Standards for Journal Articles in Primary Qualitative, Qualitative Meta-Analytic, and Mixed Methods Research in Psychology [31]. This framework ensured methodological integrity throughout the study.

*Data adequacy*. The data was obtained from the comments described by the participants, seconds after finishing the game. Each student provided individual responses to prevent interference and ensure the authenticity of their expressions. Participants were explicitly informed that there were no right or wrong answers. Emphasis was placed on the diversity of responses, which reflected the distinct temporal strategies associated with their motor conducts. This approach aligns with established standards for reporting qualitative research in psychology, contributing to the reliability and validity of the study’s findings.

*Conclusions based on the evidence*. The findings are drawn from a meticulous analysis of the students’ texts in their literal form. The methodology employed is grounded in evidence gathered from previous research, including doctoral theses and high-impact scientific articles. These foundational studies provided a robust basis for the current investigation. All of this has allowed this study to be carried out to reveal the phenomenon of the decisional meaning of the game, which is invisible to any observer.

*Data quality control*. The content analysis underwent a meticulous six-month procedure conducted by two researchers. Two university students specializing in physical activity and sports sciences, holding a master’s degree in physical education, actively participated in this process. They were not only experts in the discipline of motor praxeology but also well-versed in prior studies related to the content analysis of qualitative data in different playing scenarios. The dataset, consisting of 90 comments, was meticulously stored in an Excel format database. Following this, each text was meticulously linked to one of the five distinct emotions. To enhance their analytical prowess, these researchers engaged in a comprehensive 40-hour training programme on content analysis, aligning with the guidelines set forth by Anguera and Blanco [39]. This training initiative facilitated the creation of a detailed reference manual outlining the criteria and components for each unit of emotional meaning.

The manual was developed through a systematic process involving (1) reduction, (2) separation, and (3) synthesis and grouping of emotional meaning units. These units underwent iterative deductive and inductive reviews by the three researchers, resulting in a final version. Subsequently, a collaborative analysis of the initial 50 comments was conducted by the two researchers, followed by individual analyses of the subsequent 50 comments. Any disagreements were deliberated until a unified result was achieved, ensuring complete inter-evaluator agreement. This iterative process was repeated until the analysis of all comments was concluded. The units of meaning in the manual underwent multiple deductive and inductive reviews, leading to a definitive version. The researchers conducted joint analyses of the first 50 comments, followed by separate analyses of the next 50 comments. Discrepancies were discussed and resolved to ensure unanimous agreement between evaluators. This cycle continued until the analysis of all comments was finalized.

To gauge observer agreement, Cohen’s kappa coefficient was employed, measuring stability and objectivity. The values ranged from .86 to .91 in the content analysis of the initial 50 comments and from .90 to .96 in the subsequent content analysis.

The quality of the interpretative research was supported by the criteria of credibility and confirmability of the results through the triangulation of methods and researchers and external verification through the critical judgement of peers in the motor action research group (GIAM) [28, 34].

*Phase 3*. *Statistical analysis of quantitative data on motor conducts*. Following the content analysis of comments, three quantitative variables were identified for each role in the Elbow Tag game.

Cat. For the Cat role, the predominant temporal strategy was categorized into three options: 1 Quickly go for the Mouse; 2 take some time before trying to capture the Mouse; 3 both strategies depending on the play.

Mouse. Similarly, for the Mouse role, the temporal strategy options were related to going to the chosen Pitcher: 1 Quickly go to the chosen Pitcher; 2 take some time before going to the chosen Pitcher (play with the Cat); 3 both strategies depending on the play.

The following action consisted of quantifying [39] these variables and categories in order to assign numerical values to the data conceived as non-numerical. This action facilitated the statistical analysis to deepen the interpretation of the phenomenon studied.

It was studied whether there were differences in the presence of these variables in both versions. To do this, the associative power of the variables was addressed by Crosstab command (*p* < .05) [40] with adjusted residuals (ARs) >1.96 or <−1.96, as well as their respective effect sizes (Cramer’s V test). The following values were used to interpret the effect sizes: 0.10 = small effect, 0.30 = medium effect, and 0.50 = large effect [41].

In this study, an analysis of the intensities of emotional states, both positive and negative, was carried out using the GES-II scale, in the roles of Cat, Mouse and Pitcher. Specifically, significant differences in these values between versions 1 and 2 of the Elbow Tag game were explored through the Mann-Whitney U Test with a significance level of *p* < .05.

Additionally, Independent median tests were also conducted, presenting asymptotic significances at *p* < .05.

## 3. Results

### 3.1 Study 1. The temporal plot of motor behaviours

#### 3.1.1. Decisional dimension (cyclical time)

*3*.*1*.*1*.*1*. *The social time of the motor behaviours of the players in each of the three roles (G*, *R*, *and C) and their sub-roles during the V1 and V2 modalities)*. In both versions of the Elbow Tag game, the total time spent in different roles demonstrates a consistent pattern. The internal logic of the game indicates that players spend the longest time in the Pitcher role (M = 2,67; SD = 6,60), followed by the Cat role (M = 1,22; SD = 0,58), and finally, the Mouse role (M = 1,16; SD = 0,56). This trend is observed in both versions. However, there are some nuances between the two versions. In version 1, players spend more time in the role of Elbow Tag (M_1_ = 3,39; SD_1_ = 7,06) compared to version 2 (M_2_ = 3,22; SD_2_ = 6,12),when considering Pitcher role as an example, it was observed that the time intervals were only slightly longer than 3 seconds. This was because transitions between roles were not calculated exclusively, but changes in the state of any variable were considered. When a variable changed its previous state, it was regarded as a sequentially differentiated state. Therefore, it is essential to understand that the 3-second margins mentioned concerning the Pitchers refer to the permanence of all the variables that made up the Temporal Pattern Analysis (TPA) database. Conversely, in mode 2, players allocate more time to the roles of Cat (M_2_ = 1,27; SD_2_ = 0,67; M_1_ = 1,18; SD_1_ = 0,49) and Mouse (M_2_ = 1,18; SD_2_ = 0,67; M_1_ = 1,14; SD_1_ = 0,42).

*3*.*1*.*1*.*2*. *Decisional dimension (cyclical time)*. *The cyclic time (transition time when changing roles) of the motor behaviour of the players in the V1 and V2 modalities*: *The cyclical transition time between roles in the two versions*. In the cross-tabular analysis, the relationship between transition time and modalities V1 and V2 was explored. The Chi-Square analysis produced significant results, indicating a substantial association between the variables. Specifically, the Pearson Chi-Square value was 201.343 (*p* < .001), suggesting a highly significant relationship.

The examination of transition times was conducted meticulously, with observations recorded second by second. Each time a transition occurred, whether within the same role or between roles, it was noted.

*Staying in the same role*. The analysis revealed interesting findings regarding the duration of players in specific roles across different modalities (V1 and V2) in the studied game:

Cat Role (CC): In V1, players remained in the Cat-Cat role (CC) for a longer duration (299 seconds) with a positive residual fit of 5.3. In V2, the duration decreased to 194 seconds with a negative residual fit of -5.3.

Mouse Role (MM) and Pitcher Role (PP): No significant differences were observed between V1 and V2 for the Mouse-Mouse (MM) and Pitcher-Pitcher (PP) roles, with residual adjustments (ar) staying below 1.9 or -1.9. For PP, similar results were found in V1 (1506) and V2 (1582), indicating consistency across modalities. Similarly, for MM, the results were similar in V1 (227) and V2 (245).

Expected Values and Residual Fits: The expected values for the Cat-Cat role in both versions were 243.3 and 249.7, respectively. A positive residual fit of 5.3 in V1 suggests an observed frequency higher than expected. For the Conflict (CM) transition, 47 cases were recorded in modality 1 and 34 in modality 2, with residual adjustments of 1.6 and -1.6, respectively.

Role Change from Cat to Mouse (CM). The trend was similar in both modalities V1 (47) and V2 (34).

Change from Mouse to Cat (MC). V2 (121, *ar* = 5.4) caused more changes than V1 (50, *ar* = -5.4)

Changed from Mouse to Pitcher (MP). V1 (79, *ar* = 8.7) elicited more changes than V2 (23, *ar* = 8.7) elicited more changes than V2.

Change from Pitcher to Mouse (PM). V1 (82, *ar* = 9.8) elicited more changes than V2 (0, *ar* = -9.8) elicited more changes than V2.

Change from Pitcher to Cat (PC). V2 (85, *ar* = 9.7) elicited more changes than V1 (0, *ar* = -9.7).

*The cyclical transition time between subroles in the two versions*. The analysis of total time spent in the Elbow Tag game across two modalities reveals interesting insights (see Table A6 in S1 Appendix, Players, following the internal logic of the game, spent the most time in the Pitcher role (*M* = 2,67; *SD* = 6,60), followed by the Cat role (*M* = 1,22; *SD* = 0,58), and the Mouse role (*M* = 1,16; *SD* = 0,56). The cross-table analysis showed the existence of significant differences (*p* = 0,41), in the distribution of time in some subroles of the three roles of Cat, Mouse and Pitcher. The effect size calculated with Cramer’s V value was found to be small (.041) in the context of the Elbow Tag game. However, despite its small magnitude, the effect size indicated a significant relationship within the Cat role, specifically in the subroles CT (Cat-Chaser) and CC (Cat-Hunter).

- Time in the sub-roles of the Pitcher role. The players spent 66.8% of the two games in the Pitcher Waiting (POH) subrole. In version 2, players tended to spend more time in this subrole (34.3%, *ar* = 1.2) than in version 1 (32.6%, *ar* = -1.2).
- Time in the subroles of the Cat role. Secondly, players spent 11% of the total time in the Chasing Cat (CT) subrole. In version 1, players tended to spend 5.7% (*ar* = 1.3) of the time in the CT subrole, while in version 2 they spent somewhat less time in that subrole (5.3%, *ar* = -1,3).
- Time in the subroles of the Mouse role. Thirdly, players tended to stay longer in the Mouse-Awayer (MX) subrole (10.2%), although no significant differences were found between both versions. Players spent little time in the MJ Mouse-Caught subrole (0.6%). In version 1, players tended to spend more time in the "MJ" subrole (0.9%) compared to version 2 (0.7%), with adjusted residual values of *ar* = 1.6 and *ar* = -1.6, respectively.

#### 3.1.2. Relational dimension (cyclical time). The number of relationships triggered by the motor behaviours of the players in both versions V1 and V2

The analysis of the Elbow Tag game’s role changes reveals interesting patterns in relationship dynamics (Table A6 in S1 Appendix). In this section, we observe the quantity of motor interactions exhibited by players based on their roles. For instance, we analyze the variety of individuals the Cat pursued and how many different people the Pitchers shared this role with, when transitioning from the role of a Mouse to a Pitcher. Due to the text’s constrained length, our focus here is on tallying the overall count of motor interactions across the three roles.

Most sequences generated a low number of relationships (LR = 1; 67.4%), followed by a slight number of relationships (SR = 2; 16%). Additionally, moderate relationships (MR = 3–4; 13.4%) occurred in a lower percentage, and high relationships (HR = >5; 4.7%) were less frequent.

The statistical analysis revealed highly significant differences (*p* < .001), supported by Cramer’s V coefficient of 0.084. This analysis focused on comparing observed frequencies with expected frequencies concerning the number of relationships with other individuals in the two versions of the Elbow Tag game. Version 1 recorded more than twice as many high ratios (HR): V1 (*n* = 106, *ar* = 4.7), compared to version 2: V2 (n = 50, *ar* = -4.7). It also showed a greater number of mid-level relationships (MR) V1 (*n* = 339, *ar* = 2.5), compared to version 2: V2 (*n* = 288, *ar* = -2.5). This regularity was in the opposite direction in the relationships of low values that were more present in version V2 (*n* = 1668, *ar* = 4.2), compared to version V1 (*n* = 1493, *ar* = -4.2).

These results show that version 1 of the Elbow Tag yielded a greater number of motor interactions (high and moderate relationship range) than version 2 (low relationship range). The statistical analysis, substantiated by Cramer’s V value, reinforces the significance of these observed differences.

#### 3.1.3. Organic dimension (cyclical time)

*3*.*1*.*3*.*1*. *The physical effort (magnitude vector*, *Vm*^*2*^*) generated by participation in the roles of Cat and Mouse in the V1 and V2 modalities*. Table A7 in S1 Appendix, presents the distribution of the Vm^2^ values (Magnitude of Effort Value) concerning versions V1 and V2 of the Elbow Tag game.

The overall observation reveals that the "L" (Light) category is the most prevalent, constituting 32% of the total. Following closely, the "V" (Vigorous) category accounts for 27.9%, while the "S" (Sedentary) category holds 25.5%, and the "M" (Moderate) category comprises 14.6% of the total.

The statistical analysis found no significant differences (*p* = .271) in the distribution of Vm^2^ values between game versions for the Elbow Tag game modalities. Both game versions exhibited similar Vm^2^ distributions, and Cramer’s V value supports the absence of a significant association between these variables.

Cat Role. For the "Cat (C)" role: Significant differences (*p* < .001) were observed in the distribution of Vm2 values in relation to the two versions of the game.

Version 2 caused a greater presence of low effort values S (Sedentary; *ar* = 4.8) and L (Light; *ar* = 2.9), than version 1. This regularity was reversed in the values of higher effort V (Vigorous; *ar* = 5.1) that were more present in version 1.

Role Mouse (M). Significant differences (*p* < .001) were found in the distribution of Vm2 values in relation to the two versions of the game. The results showed an inverse orientation to that of the Cat role.

Version 1 caused a greater presence of low effort values S (Sedentary; *ar* = 5.2) and L (Light; *ar* = 2.6), than version 2. This regularity was reversed in the values of higher effort V (Vigorous; *ar* = 4.8) that were more present in version 2

*3*.*1*.*3*.*2*. *Physical effort (steps) generated by participation in the roles of Cat and Mouse in the V1 and V2 modalities*. No significant differences (*p* = .486) were found in the distribution of steps between the two versions of the game. Cramer’s V value (.023) further supports the absence of a significant association between the variables. Moreover, the analysis of adjusted residual values indicates no significant discrepancies between observed and expected values in different categories of the steps variable concerning the two game versions.

The "LS" (Low) category was the most common, representing 82% of the total, followed closely by the "SS" (Slight) category with 13.3%. The "MS" (Moderate) and "HS" (High) categories had a lower incidence, with 4.7% and 0.1% of the total, respectively.

If the data is analysed, according to the roles, the following findings are observed.

Role Cat (C). The analysis revealed significant differences (*p* < .001) in the distribution of Steps values for the Cat role (C) concerning the two versions of the game. Interestingly, the results indicate an inverse orientation compared to the Cat role.

Version 1 caused a greater presence of low values of SS steps (Slight; *ar* = 2.4) and MS (Moderate; *ar* = 3.5), than version 2. This regularity was reversed in the values of the number of LS steps (Low; *ar* = 4.5) that were more present in version 2.

Role Mouse (M). The analysis indicates significant differences (*p* = .002) in the distribution of Steps values for the Mouse role (M) concerning the two versions of the game. Interestingly, the results suggest an inverse orientation compared to the Cat role.

Version 2 caused a greater presence of low values of steps SS (Slight; *ar* = 2.3) and MS (Moderate; *ar* = 2.2), than version 1. This regularity was reversed in the values of LS (Low; *ar* = 3.7) which were more present in version 2.

**Fig 5:**
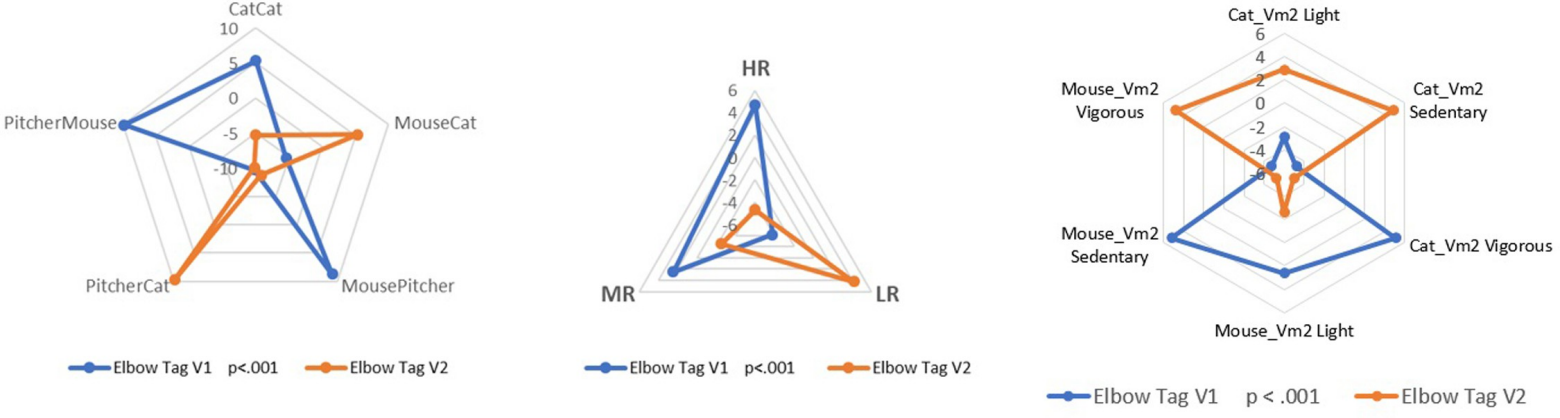
Multidimensional comparison of social time based on adjusted residuals in Elbow Tag game (V1_V2). Based on Table A8 in S1 Appendix. The cyclic time of transition by roles in V1 and V2. Crosstab and adjusted residuals. **Based on Table A9 in S1 Appendix.** Relational cyclic time in versions V1 and V2. Crosstab and adjusted residuals. **Based on Table A10 in S1 Appendix.** Comparison of the cyclic time of physical effort (vector magnitude Vm^2^) by roles of Cat and Mouse in V1 and V2. Crosstab and adjusted residuals.

The following Fig 5 facilitates the visualization of significant cross-table differences observed in social time in the V1 and V2 versions. This is based on motor behavior in the transitions between subroles (cognitive dimension), the number of motor interactions (social dimension), and the physical effort (vm^2^) (organic dimension).

#### 3.1.4. Multidimensional cyclical time (360º). Multidimensional temporal variables predicting the number of motor interactions in V1 and V2 of the Elbow Tag game

This article has considered studying the social time of motor behaviours from cognitive, relational and organic dimensions. Given the importance of promoting relational physical education, we have decided to choose the number of motor interactions as the dependent variable in this section. Thus, when predicting or explaining the number of relationships (dependent variable) derived from the Elbow Tag game, the classification tree gave different predictive power to the following variables: a) subroles, b) version of the game, c) the magnitude of effort, d) playing field, e) a number of players (all of them independent variables).

The tree showed that the subroles are the first variable to be considered to predict the number of motor interactions in the pitcher game. Furthermore, it was observed that not all the subroles contributed the same distribution in the number of interactions; that is, significant differences were found (*p* < .001; X^2^ = 627.886; *df* = 9) in the number of relations originating from the subroles referred to the three Roles. a) The majority of cases (66.8%) corresponded to the role of the Pitcher (node 4, POH, subrole: Pitcher in waiting); b) In the second place, the Mouse (node 3) originated 15.4% of the cases, in the three subroles: Mouse Pursued (MX), Mouse-Eastener (MC), and Mouse Caught (MJ). Next, c) the Cat recorded 11% of the cases (node 1, Cat Pursuer CT). Finally, a node (2) was also observed, which grouped 6.7% of the cases through the subroles referring to Cat (Cat-Catcher, CC), to Pitcher (Pitcher-Unfastener, PL) and to be in Conflict (CF). The Mouse subroles (node 3) originated 100% of a low number of motor interactions (LR). The low number of relationships predominated in all roles, although the highest number of relationships (HR) originated in the Pitcher subrole (POH, *n* = 137; 4.4%), followed by the Cat (CT, *n* = 18; 3.5%).

The tree established two major branches from the variables, Cat-Chaser (CT) (nodes 1, 5, 6, 11 and 12) and Pitcher-in-Hold (POH) (nodes 4, 7, 8, 9, 10, 13, 14, 15 and 16).

*Branching from the Cat role*. When players adopt the Cat-Chaser (CT) subrole (node 1), the number of motor interactions will depend mainly on the game version. Significant differences (*p* < .001; X^2^ = 267.840; *df* = 3) were found in the number of relationships originating from this role in version 1 (node 5) and version 2 (node 6). While in version 2, the Chasing Cats always created a low number of relationships (LR = 100%), in mode 1, the number of low (LR), light (sr) and moderate (MR) relationships reached similar values (from 27.5% to 34.9%). In version 1, 6.7% of high ratios (HR) were also recorded.

Finally, in this branch, for V1, the tree identified the variable number of steps to predict the number of relationships that originated in the role of the chasing cat. Significant differences were found (*p* < .001; X^2^ = 20.181; *df* = 3) in the number of relationships according to the number of steps. When the number of steps is very low (LS, node 11), a low number of relationships predominates (LR, 46.5%), while when the number of steps is higher (SS, MS; node 12), the number of relationships increases (SR = 42.1%).

*Branching from the Cat role*. When players adopt the Cat-Chaser (CT) subrole (node 1), the number of motor interactions will depend mainly on the game version. Significant differences (*p* < .001; X^2^ = 267.840; *df* = 3) were found in the number of relationships originating from this role in version 1 (node 5) and version 2 (node 6). While in version 2, the Chasing Cats always created a low number of relationships (LR = 100%), in mode 1, the number of low (LR), light (SR) and moderate (MR) relationships reached similar values (from 27.5% to 34.9%). In version 1, 6.7% of high ratios (HR) were also recorded.

Finally, in this branch, for V1, the tree identified the variable number of steps to predict the number of relationships that originated the role of the Cat chaser. Significant differences were found (*p* < .001; X^2^ = 20.181; *df* = 3) in the number of relationships according to the number of steps. When the number of steps is very low (LS, node 11), a low number of relations predominates (LR, 46.5%), while when the number of steps is higher (SS, MS; node 12), the number of relations increases (SR = 42.1%).

**Fig 6:**
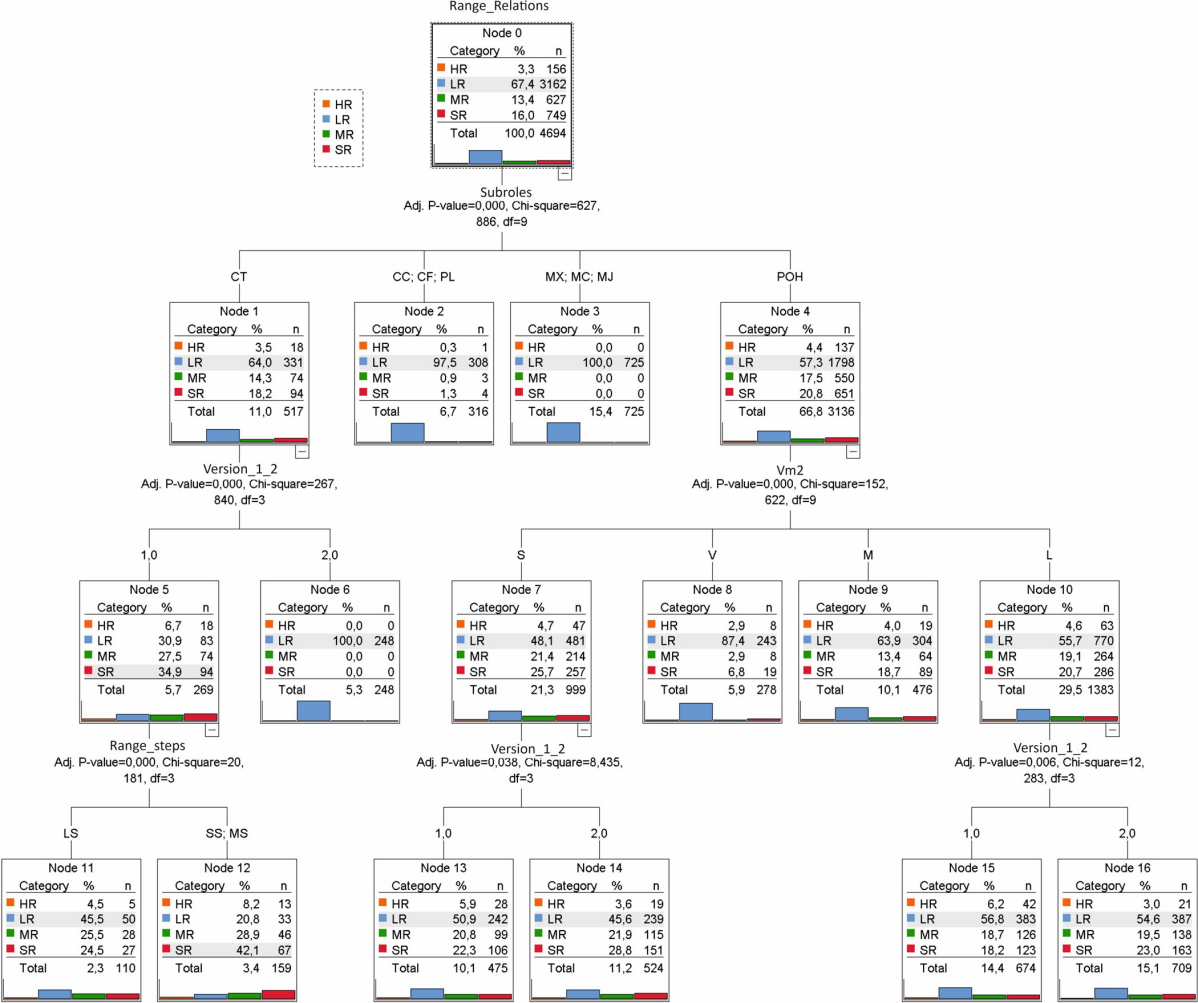
Classification tree on the temporal variables (organic, decisional and relational) of the players’ motor behaviours, with greater predictive power for the number of relationships between players that originate the V1 and V2 versions of the Elbow Tag game. Note. Subroles: CC Cat-Catcher; CF Conflict; CT Cat-Purser; MC Mouse-Easterner; MJ Mouse-Caught; MX Mouse-Pursued; POH Pitcher-Waiting; PL Pitcher-Unfastener; Steps: Sedentary: Low LS (0–1); Slight SS (2); Moderate MS (3) High HS (4); Effort magnitude value (Vm^2^): Sedentary: S (0–2 CPS); Light L (<2-34CPS); Moderate M >34–100 CPS; Vigorous V (>100CPS). Relationships: LR (Low Relationships = 1); SR (Slight Relationships = 2); MR (Moderate Relationships = 3–4); HR (High Relationships = >5).

*Branching from the role of the Pitcher*. When players are in the subrole Pitcher in waiting (POH, node 4), the number of relations they originate depends on the effort made (vector magnitude of physical effort, Vm^2^). Most of the time (*n* = 999; 21%), the effort is sedentary (S) (node 7) or Light (L) (*n* = 1383, 29.5%). When the effort was sedentary, the V1 and V2 versions recorded significant differences in the number of motor interactions (*p* = .038; X^2^ = 8.435; *df* = 3). Although the number of relations of the Pitchers was very small, V1 outperformed V2 (V1:LR = 50.9%; V2: LR = 45.6%). Secondly, the range of light ratios was higher in V2 (V1: SR = 24.5%; V2: 28.8%). Finally, in V1, the pitchers interacted with more people (RH) than in V2 (V1: RH = 5.9%; V2: RH = 3.6%). When the Vm^2^ effort was light (L, node 10), differences were also found between the V1 and V2 versions (*p* = .006; X^2^ = 12.283; *df* = 3), following the same pattern as in the previous case (Fig 6 and Table A11 in S1 Appendix).

#### 3.1.5. Multidimensional cyclical time. Multidimensional T-Patterns (360º) (decisional, relational and organic) of the motor behaviours in the V1 and V2 versions

The study identified 559 multidimensional T-Patterns (TP) (decisional: sub-roles, relational: number of interactions and organic: physical effort Vm*2* and number of steps), 209 TPs in version 1 and 350 TPs in version 2. In addition, TPs were identified six levels of complexity, considering the number of strategic TP links (see S1 Appendix).

Complexity 1 (1 link): 216 T-Patterns (*n* = 86 in V1; *n* = 130 in V2);

Complexity 2 (2 links): 96 T-Patterns (*n* = 86 in V1; *n* = 130 in V2);

Complexity 3 (3 links): 31 T-Patterns (*n* = 31 in V1; *n* = 65 in V2);

Complexity 4 (4 links): 21 T-Patterns (*n* = 6 in V1; *n* = 15 in V2);

Complexity 5 (5 links): 8 T-Patterns (*n* = 0 in V1; *n* = 8 in V2);

Complexity 6 (6 links): 2 T-Patterns (*n* = 0 in V1; *n* = 2 in V2)

The cross tables showed significant differences in 18 TPs of multidimensional strategies, of which 11 TPs were more present in V1 and 7 in V2 (look at Table A12 in S1 Appendix).

The number of times that each category of the different PD variables appeared in the 6 levels of complexity was also identified. 67 chains were recorded, with the predominance of the following categories for each variable and dimension of motor behaviours:

*Cognitive dimension*. Mouse-Pursued (MX): 22 links at all levels of complexity; Cat-Pursuer (CT): 22 links at all five levels of complexity; Mouse-Easterner (MC): 9 links at 6 levels of complexity; Pitcher-Unfastener (PL) 7 links in 5 levels of complexity; and Mouse Caught (MJ): 3 links in 2 levels of complexity (see Table 1).

**Table 1:** Cognitive dimension based on T-Patterns’ complexity.

Decisions (Cognitive Dimension)									
Role	Subroles		Complexity 1	Complexity 2	Complexity 3	Complexity 4	Complexity 5	Complexity 6	Total
**PITCHER**	POH	Waiting	0	0	0	0	0	0	0
	PL	Unfastener	2	1	2	1	1	0	7
**CAT**	CT	Purser	6	8	4	3	1	0	22
	DC	Catcher	1	1	1	0	0	0	3
**MOUSE**	MX	Pursued	6	6	4	4	2	1	22
	MC	Easterner	1	3	2	1	1	1	9
	MJ	Caught	2	1	0	0	0	0	3
**CONFLICT**	CF	Conflict	0	0	0	0	0	0	0
**TOTAL**			18	20	13	9	5	2	67

*Relational dimension*. Low (L): 62 links at all levels of complexity; Moderate (MR): 4 links in 3 levels of complexity; Slight (SR): 1 link in a complexity level (see Table 2).

**Table 2:** Relational dimension based on T-Patterns’ complexity.

Relationships (Relational Dimension):									
	Relationship		Complexity 1	Complexity 2	Complexity 3	Complexity 4	Complexity 5	Complexity 6	Total
	LR	Low	16	18	12	9	5	2	62
	MR	Light	1	0	0	0	0	0	1
	MR	Moderate	1	2	1	0	0	0	4
	HR	High	0	0	0	0	0	0	0
TOTAL			18	20	13	9	5	2	67

*Organic dimension (Physical effort*, *Vm*^*2*^*)*. Vigorous (V): 47 links at all levels of complexity; Sedentary (S): 7 links in 5 levels of complexity; Light (L): 7 links in 5 levels of complexity; Moderate (M): 6 links in 4 levels of complexity; Slight (SR): 1 link in a complexity level (see Table 3).

**Table 3:** Organic dimension (in Vm^2^) based on T-Patterns’ complexity.

Physical Effort (Vector magnitude Vm^2^) (Organic Dimension)									
	Vm2		Complexity 1	Complexity 2	Complexity 3	Complexity 4	Complexity 5	Complexity 6	Total
	S	Sedentary	1	3	1	1	1	0	7
	L	Light	3	1	1	1	1	0	7
	M	Moderate	2	2	1	1	0	0	6
	V	Vigorous	12	14	10	6	3	2	47
TOTAL			18	20	13	9	5	2	67

*Organic dimension (Steps)*. Low (LS): 40 links at all levels of complexity; Slight (SS): 19 links in ALL 6 levels of complexity; Moderate MS): 8 links in 4 levels of complexity (Table 4).

**Table 4:** Organic dimension (in steps) based on T-Patterns’ complexity.

Physical Effort (steps) (Organic Dimension)									
	Steps		Complexity 1	Complexity 2	Complexity 3	Complexity 4	Complexity 5	Complexity 6	Total
	LS	Low	11	13	7	5	3	1	40
	SS	light	6	4	3	3	2	1	19
	MS	Moderate	1	3	3	1	0	0	8
	HS	High	0	0	0	0	0	0	0
TOTAL			18	20	13	9	5	2	67

The analysis of cross tables, as detailed in Table A12 of S1 Appendix, has brought forth noteworthy findings regarding T-Patterns in the traditional Elbow Tag game versions (V1 and V2). The results indicate statistically significant differences, underscored by a low p-value (*p* < .001) and a substantial Effect Size of 0.46 depending on the level of complexity. Specific differences were contrasted between the two versions of the game in complexity 1 (*p* < .001; ES = 0.45), complexity 2 (*p* = .001; ES = 0.45) and complexity 3 (*p* = .025; ES = 0.49). On the other hand, at complexity level 4 no differences were evident, while at complexity levels 5 and 6 no statistical evidence could be found, due to the lack of T-Patterns on the part of V1.

Within each level, the largest positive and/or negative asymmetries found were selected, following the Residual Adjustments (RA). Thus, at level 1 of complexity V1 surpassed (*n* = 9, *ar* = 3.8) V2 (*n* = 0, *ar* = -3.8) through the T-Pattern (mj_v_ls_lr). Also, at level 2 of complexity based on the T-Pattern (mj_v_ls_lr) V1 exceeded (*n* = 4, *ar* = 2.5) V2 (*n* = 0, *ar* = -2.5). However, at level 3 a triple tie was found between V1 and V2 with positive or negative ar. V1 surpassed (*n* = 2, *ar* = 2.1) V2 in the T-Pattern (cc_v_ls_lr), as did the T-Pattern (mc_v_ms_lr) with V1 (*n* = 2, *ar* = 2.1) (*n* = 0, *ar* = 2.1). On the other hand, the T-Pattern (mc_v_ss_lr) was mainly attributed to V2 with 19 frequencies (*n* = 3, *ar* = -2.1) (*n* = 19, *ar* = 2.1). Already at level 4 of complexity, V1(*n* = 2, *ar* = 2.4) again surpassed V2 (*n* = 0, *ar* = -2.4) through the T-Pattern (mx_m_ls_lr). Finally, the nonexistence of T- stands out. Patterns attributed to V1 in complexities 5 and 6, but it would also be notable to show the predominance of the Mouse in T-Patterns found in these last two levels of complexity, since of the 7 T-Patterns found, 5 are formed by the development of the Mouse role (*n* = 3 Mx and *n* = 2 Mc).

### 3.2 Study 2. The temporal plot of motor conducts

#### 3.2.1. The temporal strategies of the motor conducts in the roles of Cat and Mouse, expressed by the players in V1 and V2

*3*.*2*.*1*.*1*. *Temporary Cat strategies*. The cross-table technique showed significant associations (p = .003) when comparing the two versions. In Cat modality 1, the strategy of going quickly after the Mouse (strategy 1) was used more times than in version 2 (V1: n = 78; ar = 3.2; V2: n = 54; ar = -3.2). The strategy of taking some time before trying to capture the Mouse (strategy 2) was more present in modality 2 (V1: n = 8; ar = -2.3; V2: n = 20; ar = 2.3). No differences were found between V1 and V2 in the expected values in the mixed strategy (number 3).

**Fig 7:**
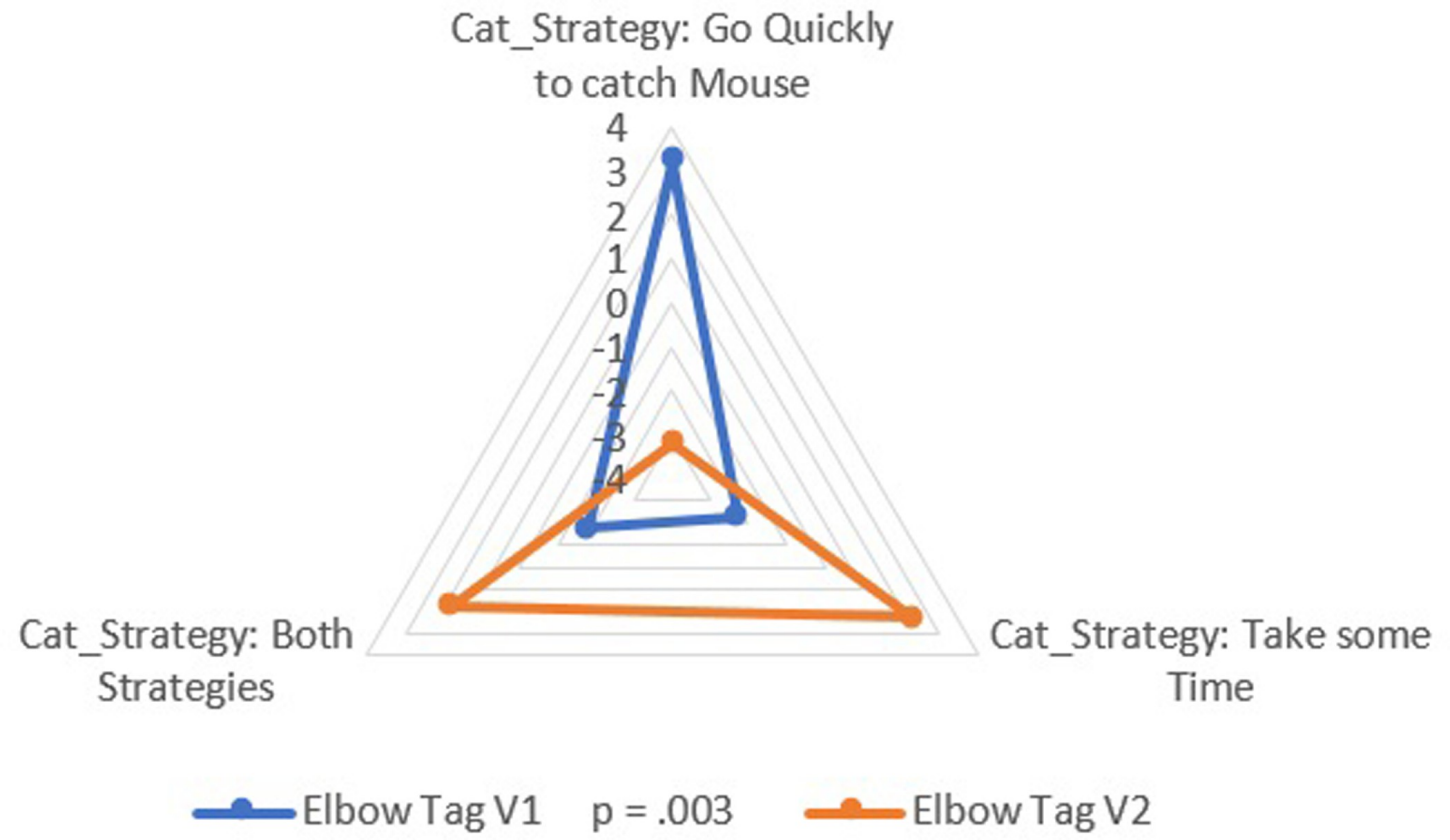
Temporal strategies used by Cat in V1 and V2 through adjusted residuals. Crosstab and adjusted residuals.

The following Fig 7 shows the significant differences in the temporal strategies of the Cat’s motor conducts when comparing the two versions in the Elbow game.

*3*.*2*.*1*.*2*. *Temporary mouse strategies*. The cross-tabulation technique (Table A13 in S1 Appendix) did not observe significant differences (*p* = .560) between the two versions, in the 261 cases (*n* = 29 in V1; *n* = 132 in Vs) corresponding to the three types of temporal strategies used by the Mouse. Strategy 1: Quickly go to the chosen Pitcher; strategy 2 Take some time before going to the chosen Pitcher. Play with the cat; Strategy 3. Use both strategies depending on the situation.

#### 3.2.2. Emotional states of motor behaviours expressed by the players, in versions V1 and V2

The descriptive analysis showed the emotional intensity that the players experienced when starring in the three roles in V1 and V2.

The emotion joy reached the highest values in the three roles: Mouse Joy (V1 *M* = 5,31, *SD* = 1.631; V2 *M* = 5.23, *SD* = 1.611); Cat Joy (V1 *M* = 4.44, *SD* = 1.800; V2 *M* = 4.87, *SD* = 1.644) Pitcher Joy (V1 *M* = 4.59, *SD* = 1.823; V2 *M* = 4.46, *SD* = 1.832).

This study employed the non-parametric Mann-Whitney U test to assess the intensity of five emotions (joy, anger, fear, rejection, sadness) in two versions. Significant differences (*U* = 11.204, *p* = 0.34) were found specifically in the emotion of joy within the Cat role. Players with the Cat role in Version 1 reported lower joy values (*M* = 4.44, *SD* = 1.800) compared to Version 2 (*M* = 4.87, *SD* = 1.644) of the game.

**Fig 8:**
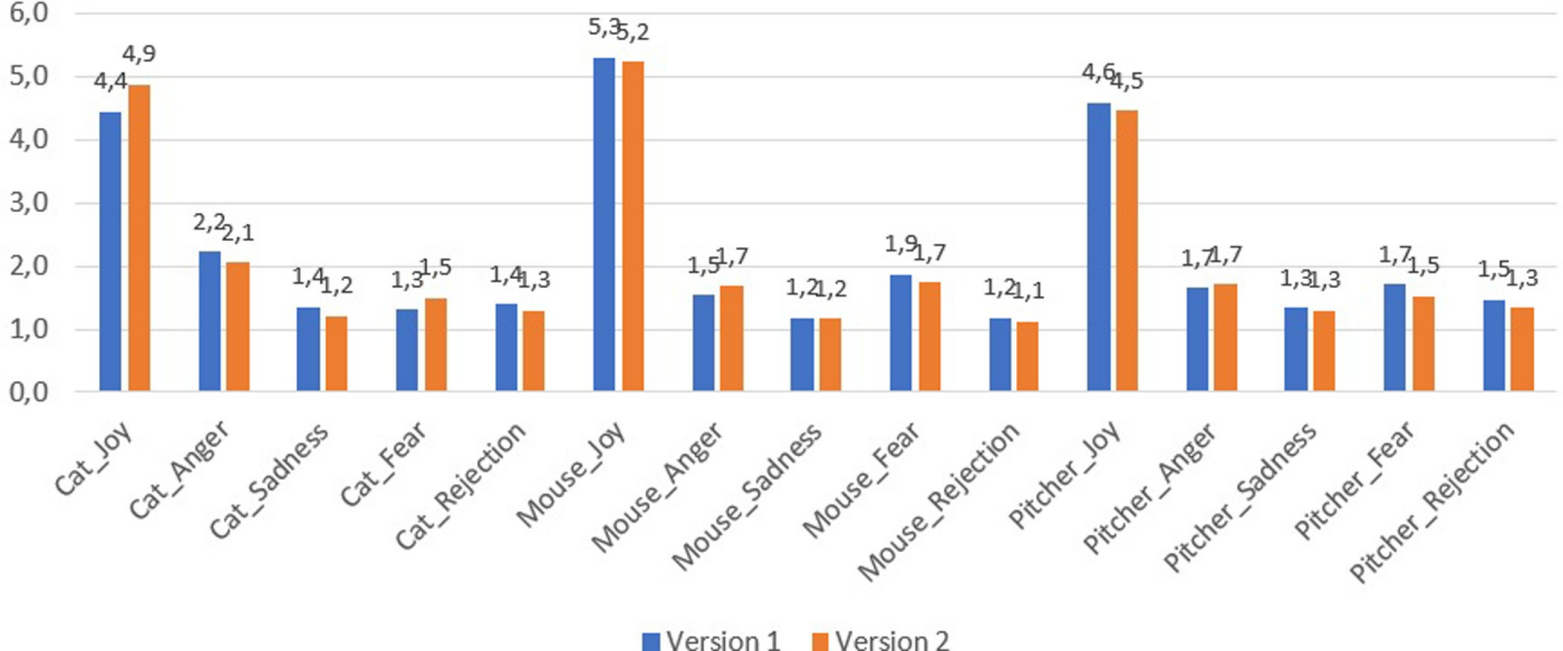
Intensity in the five basic emotions in the three Roles for V1 and V2.

Regarding negative emotions, specific trends were identified, though they did not yield statistically significant differences (see Fig 8 and Table 5, Table A14 in S1 Appendix).

**Table 5:** Intensity in the five basic emotions in the three for V1 and V2.

	Cat		Mouse		Pitcher	
	V1	V2	V1	V2	V1	V2
joy	4.44 ± 1.80	4.87 ± 1.6	5.31 ± 1.63	5.23 ± 1.61	4.59 ± 1.82	4.46 ± 1.83
anger	2.23 ± 1.58	2.06 ± 1.43	1.54 ± 0.99	1.68 ± 1.78	1.65 ± 1.30	1.71 ± 1.31
sadness	1.35 ± 0.89	1.21 ± 0.64	1.18 ± 0.54	1.16 ± 0.58	1.34 ± 0.89	1.29 ± 0.68
fear	1.31 ± 0.94	1.49 ± 1.18	1.86 ± 1.40	1.74 ± 1.28	1.71 ± 1.23	1.53 ± 1.1
rejection	1.39 ± 0.97	1.30 ± 0.90	1.18 ± 0.58	1.13 ± 0.41	1.46 ± 1.11	1.34 ± 0.89

Cat Role: V1 exhibited higher intensity values than V2 in Anger, Sadness, and Rejection. Fear was lower in V1 compared to V2.

Mouse Role: V1 recorded more intense values than V2 in Fear and Rejection.

Equal values were observed in Sadness. V2 induced more intense Rage values in V1.

Pitcher Role: V1 caused more intense values than V2 in Fear and Rejection.

Equal values were noted in Anger and Sadness. The most intense negative emotions were observed in Cat Rage (V1 *M* = 5.3; V2 *M* = 5.2), followed by Mouse Fear (V1 *M* = 1.9; V2 *M* = 1.7) and Pitcher Rage (V1 *M* = 1.7; V2 *M* = 1.7).

**Fig 9:**
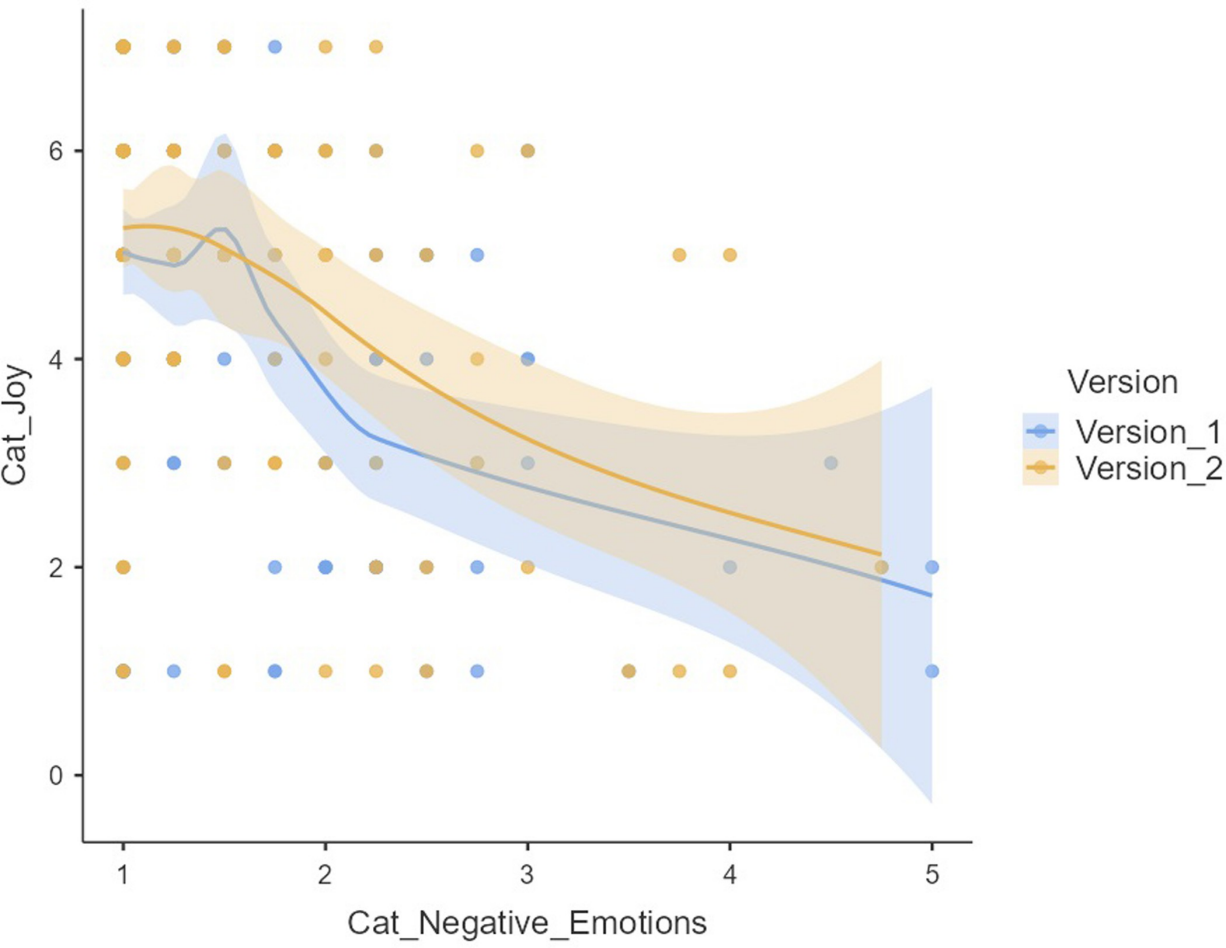
Average values of the positive and negative emotional levels of players in version_1 versus Version_2.

Fig 9 visually complements the pattern of positive and negative emotions exhibited by players in the role of Cat across the two versions, aligning with the insights presented in Fig 6. Notably, Cat players predominantly encountered more pronounced joy values in V2 (*p* = .34) compared to V1. Additionally, there is an observable trend towards recording heightened values in V1 for negative emotions.

## 4. Discussion

This research aimed to uncover the process of learning social time in the two versions of the Elbow Tag game.

This article presents two studies, referring to the temporal plot associated with motor behaviours (study 1) and motor conducts (study 2) that originate in the Elbow Tag game. The theoretical foundation is based on motor praxiology [13]. It is based on the premise that the internal logic of the Elbow Tag game contains a singular temporal plot that the players interiorize by putting into action the system of role changes. The Elbow Tag game acts as the main independent variable of the subjective time experienced by the players through their motor behaviours and motor behaviours (dependent variables). The evidence confirms the influence of the game and the staging of its rules on the motor conduct of its participants, in line with the findings of previous research initiated by Parlebas [13, 16, 19] and researchers in motor praxeology. The motor behaviours and the meaning given to them by the players (motor conducts) are filtered by the game’s internal logic [27].

The authors of this article have decided to develop the discussion section by analysing the results obtained in both studies in a systemic and unitary way. We proceed to relate the systemic analysis of the Elbow Tag game to the analysis of the person as a whole. In this case, through the multidimensional analysis of the temporal plot that integrates the motor behaviours and their significant organisation, that is to say, their motor conducts (strategic and emotional meaning). In this way, we follow a coherent discourse to promote a motor conduct pedagogy based on scientific evidence.

The findings provide compelling to reveal the temporal structure of this game. The external observation (motor behaviours) and the temporal implication (actions meaning; therefore base on) (motor conducts) greatly assist in understanding the process of social time construction.

The first contribution of this study is the use of temporal units to decipher the subjective time by participants. These units go beyond the use of an external measure, such as considering the 8 minutes or 480 seconds that each game lasted. This external time measure is enhanced by the use of internal time units of the game, referring to the players’ motor behavior (e.g., time spent in each role and sub-role; number of role changes; number of motor interactions; time spent in physical effort). Furthermore, these internal time measures are reinforced bolstered by analyzing the significance each individual (person) attribute to their temporal strategies and emotions (motor conduct).

### 4.1. The system of role changes. Key to decrypting the game’s temporal singularity

When observing a group of students participating in the Elbow Tag game, one often observes motor actions that make little sense, confusing relationships and many doubts in decision-making. However, the apparent chaos observed on the surface of the playing field rests on a solid and deep order that establishes the internal logic of this game, primarily through its system of role changes [13, 15]. It is an operational model (called universal by motor praxeology) that represents the basic structure of the functioning of this game [16]. Moreover, this model is the bearer of a unique internal logic.

The subjective temporal experience lived by the actors is constructed by putting into action a network of original role changes. The difference between the two versions of the Elbow Tag game may seem very small; only one rule is modified (Elbow Tag player will go out with the role of Cat instead of the role of Mouse). However, this slight modification radically transforms the role change system and the game’s temporal sequence logic.

The players in this game must appropriate the cyclical, open-ended time that is recurrently re-produced in each sequence of actions. This process of understanding and applying the temporal laws of the game presents itself as a complicated challenge due to the relational complexity of the game (system) and the polyhedral nature of the motor conducts (actor). "The systemic traits of the internal logic legitimise the player’s action, and it is the player’s action that allows the system to exist. The player and the system go hand in hand, each giving meaning to the other "p. 58 [16].

Immersion in this game offers a unique temporal experience and plays an educational role by influencing the social construction of playtime. This phenomenon is achieved by intricately connecting two indissoluble realities: the system of role changes (internal logic) and the actor (temporality of motor conduct) [13].

The Elbow Tag game acts as a living laboratory of relationships, as a miniature society [13, 19], where temporal learning is constructed in an affectively safe, playful environment. Physical time, measured with objective seconds or minutes, is transformed into psychological time, experienced through subjective units such as emotions, relationships, decisions or physical effort. This game is experienced as a playful ritual [6, 13, 33] in which players are forced to play a plot around three specific roles: Cat, Mouse and Pitcher.

In this temporary plot, the players, by adopting these roles, share relationships of power and submission; the Cat can catch and modify the role of the Pitcher; the Pitcher can decide more or less favourable situations for the Cat; moreover, the Pitcher has the power to select which Pitchers player will switch to an unfavourable role. At the same time, the players share intense emotions when faced with challenging situations, for example, when the Cat is about to be caught, or the Mouse is about to be caught, or when they unexpectedly step out of Pitcher’s comfort zone and start a sequence of actions full of uncertainty. Players are also tested in the management of physical effort, sometimes by using vigorous motor conducts to move quickly, in other circumstances by taking advantage of a more relaxed situation to read the game’s internal logic. Entering into this game implies entering into a civilising (socialising) process [1], in which people learn to educate the impulses of their psychological time (individual self-action) and adjust them to the codes of respectful behaviour established by society (external social coercion) [1].

The game’s temporal storyline is linked to an intense and meaningful experience [13]. People who describe the reasons for their emotional experience leave a record as if it were a fingerprint of their motor biography, in which emotion, relationship, decision and energy form part of their personal life experience.

*The fact of playing already brings me joy, so the reality of being a Cat or a Mouse [cognitive dimension] has no influence*. *Being at high pulses [organic dimension] and having to make decisions [cognitive dimension] by opposing you [relational dimension] is what generates the fun for me. Student 12 V1*

The game’s rules are social norms [15, 19], so, at an early age, learning to interpret the roles in a match will facilitate the acquisition of social roles in everyday life [33]. In everyday life, people will have the autonomy to make decisions (in the game, each player chooses the subroles) and perform social experiences (in the game, motor interactions), exchanging different roles, such as mother, lawyer or coach (in this game, as Cat, Mouse or Pitcher) [33].

For the educator, intervening in the education of temporality in this game implies, firstly, recognising the distinctive features of the game’s internal logic, concretised in the system of role changes in each version. Secondly, it is necessary to consider the aspects that intervene in the subjective time that each student constructs, thanks to their ludomotor intervention. Thus, the educator can know how to recognise the motor behaviours of each student, identifiable from external observation, and subsequently help to transform their motor conduct, that is to say, work on the internal meaning (generally invisible from an external approach) that facilitates a singular appropriation of the time frame of the game. This is how the educational process becomes a civilising process of the subjective time that shapes each person [1].

Let us go over the temporal plot of the game based on the findings obtained for each game’s roles.

### 4.2. The temporal plot in the Pitcher role. Observing educates

Throughout the eight minutes of the game (physical time), the players dedicate most of their time to the performance of the Pitcher in the standby role (66.8%). Being a Pitcher hardly involves motor commitment (the players are standing still). However, there are moments when each participant experiences intense moments of reflection and emotion.

*I felt quite happy because I was observing carefully what was happening [cognitive dimension], and depending on what was happening [cognitive dimension], I felt some emotions or others*. *Student 22 V2*

The game’s internal logic gives a starring role to two main actors, the Cat and the Mouse. The rest of the participants adopt the role of the Pitcher, being secondary actors in this plot or ritual.

*I enjoy watching my peers [relational dimension] as they play and implement different strategies [cognitive dimension], but I don’t enjoy it as much as running [organic dimension]*. *Student 54 V2*

The study does not identify T-patterns when the pitchers are on hold; only seven possible T-Patterns were observed when a player leaves the Pitcher (Pitcher-Unfastener). These are moments of great interest to remain active in the game from reflection in action. However, the lack of motor involvement causes negative emotions in some players, such as rejection towards this role.

*The most noticeable emotion is the rejection of the passivity of the role of the Pitcher [organic dimension] and the desire I had to play [organic dimension]*. *Student 60 V1*

The change in the role of the Pitcher has to be triggered by the intervention of another person. Some people express discomfort when they perceive that they have been in this role for too long because of the lack of motor empathy of the other participants.

*I felt rejected because the Mice didn’t hook [cognitive dimension] ever to my partner [relational dimension]; they always joined [cognitive dimension] to me, and I couldn’t get out [relational dimension]*. *Student 86 V2*

From an attentive look at this role, the players improve their ability to decipher the other players’ body signs and interpret the strategies used by the other players.

*I registered a high value of joy because you also have a good time, watching the others play [relational dimension] and seeing how they run after each other [relational dimension]*. *Student-5 V1*

It is a good moment to share some commentary with the other person next to you, sharing the role of Pitcher. These are moments of apparent motor and emotional tranquillity. However, this motor and emotional comfort is fragile and ephemeral, as everything can change quickly. Hence, the unease expressed by some players.

*I was afraid because if a mouse joined my partner [relational dimension], I would quickly have to go and catch the opponent [relational and cognitive dimension]*. *Student 64 V2*

Sometimes, the urgency of an escape is the Mouse who finds our Pitcher as a place of salvation; sometimes, a premeditated strategic decision by the Mouse can provoke the Pitcher’s exit without the players noticing it. It is necessary to be very vigilant.

*I felt fear with high intensity because I had to be constantly alert [cognitive dimension] to see if they chose my partner [relational dimension] so that I could escape [relational and cognitive dimension]*. *Student 44 V1*

Depending on the version, the player in the role of the Pitcher must act quickly to exit as a Mouse-Awayer in version 1 or as a Cat-Pursuer in version 2. Depending on the modality, the decisions will be different, and this modification in the role change will generate varied consequences. The problem is served, and some people stay still without leaving the Pitcher (they have not seen that the Mouse has joined the partner on the other side of the Pitcher); other players go with the wrong role (as a Cat) and chase when they have to escape (as a Mouse) or escape when they should be pursuing. The confusion is responsible for very unequal emotional reactions. Sometimes the actors’ bewilderment provokes laughter and comical situations that have an emotional impact on the group.

*In the Pitcher’s role, the highest emotion is also a joy because there were funny, confusing situations [cognitive dimension] with which the whole group laughed [relational dimension]*. *Student 101 V2*

This informational uncertainty, which generates the role of the Mouse over the Pitcher, explains why the accelerometers register light efforts and some steps in the Pitchers when they are in the waiting subrole. This is an indicator of the confusion caused by the role change system, coupled with an ambivalent or paradoxical motor communication network [13, 15]. In such circumstances, some people feel joy, others express sadness.

*When you came out of this role, pitcher, you didn’t know whether you were catching or running away [cognitive dimension]; that’s why I felt sadness as Pitcher*. *Student 72 V1*

### 4.3. The temporal plot in the Cat role. Different modes of role change

Internalising the ludic time of this game means integrating into each role a singular tempo-rationality with the meaning that each player gives to it. After the Pitcher role, the players of the playful plot spend more time in the Cat role, adopting the pursuer subrole (11% of the game, i.e. approximately one minute). In the first version, being a Cat allows us to assume a role that gives us power [33], as it is the only person who can turn the Mouse into a Cat.

The role of the Cat is perceived as a strong role that the students like to play. In this role, the students feel empowered as they take a large part of the active leading role in the game.

*The Cat role has more participation [organic dimension] and importance and seems to be the most fun*. *Student 4 V2*

*Joy because we all like being in this role [cognitive dimension]; it was a lot of fun and one of the most active forms of participation [organic dimension]*. *Student 49 V2*

*My emotional experience as a Cat has been one of joy, basically because it was a game where chasing someone [relational dimension] was a very exciting stimulus, and this gave me quite an excitement, i.e. I felt very active [organic dimension]*. *Student 94 V1*

However, this power is relative since, depending on how the Mouse intervenes, the Cat will have to submit his/her intervention to prolonged sequences (when the Mouse plays with the Cat and tricks him/her when he confuses him or makes him/her go to different areas of the field). At other times, his/her intervention will follow the temporal dictates of shorter sequences (when the Mouse moves slowly and manages to catch him or when the Mouse quickly joins a Pitcher). If we compare the two versions, we can see that in V1, the Cat does not always succeed in capturing the Mouse (R1) quickly. Thus, the sequence can take longer than expected, as the Cat must regularly chase several Mice (R2, R3, R4 …). In these game phases, the joy can be accompanied by a certain feeling of discomfort.

*Sometimes, you go a long time without catching the Mouse [relational dimension]; if you are 20 or 30 seconds without catching it between being tired [organic dimension], you feel happy but also a bit angry*. *Student 118 V1*

The variety of strategies and possibilities of action of the Cat, captured from external observation, explains that the Cat Pursuer originates a high number of temporal patterns (22), which give rise to up to five different T-patterns (complexity level 5) in the strategies of the players. The results indicate that the strategies are composed of multidimensional internal time (temporal links) that are integrated by the players in regularities: decisional (subroles), relational (number of interactions) and organic (magnitude of physical effort Vm^2^ and steps).

Moreover, when explaining their strategy and emotions, the content analysis of the protagonists’ testimonies confirms that each person socialises a particular temporal experience. Each person integrates the different temporal units of this subjective time with a different meaning. Decisions and emotions can respond to an organic, relational motive, a cognitive reading, or a combination of meanings.

Comments on the strategy. *Depending on who was chasing me [relational dimension] and how tired I was [organic dimension]*, *I would go straight to a pitcher [cognitive dimension]*, *or I would give myself time to play with the Cat [relational dimension] and laugh at him [emotional and relational dimension]*. *I usually played with the Cat [relational dimension]*. *Student 74 V1*

Commentary on emotion. *I felt*, *above all*, *joy because I had a lot of fun playing*, *but also anger because it depends on who we have to chase [relational dimension]; we can play for a long time without changing roles [cognitive dimension]*. *It can get tiring [organic and emotional dimension]*. *Student 58 V1*

Everything appears united, indissoluble, forming part of a complex weft that weaves the system of role changes and accompanies this contextualised subjective experience of time. We are dealing with the personalised process referring to the social construction of time [12, 33].

#### 4.3.1. Version 1. The change of role from Cat to Mouse. A merit of its own

In the first version, the Cat must accept that it cannot switch roles until it captures a Mouse. This can sometimes be difficult and involve chasing several mice (high range of motor interactions). The findings confirm this correspondence between internal logic and the consequences of the game actions; in V1, the Cat registers higher values than in V2 in: a) the time of participation in this role; b)The number of motor interactions with the Mice; c) the time spent performing actions with more intense physical Effort; d) using a larger number of steps.

The players’ testimonies explain the particularities of their temporal strategies and, simultaneously are like, fingerprints. These deeply imprinted details permeate the subjective construction of the social time of the game.

The primary strategy in the first version consists of the Cat going quickly to catch the Mouse. However, it responds to different meanings of the actors’ subjective time.

In some people this strategic motor conduct responds to an organic (energetic) meaning. Strategic time is internalised as energetic time.

*My strategy as a Cat was to move quickly towards the Mouse [cognitive dimension] because it could join a Pitcher and introduce a "new" Mouse that was not physically exhausted [organic dimension]. Otherwise, it would have difficulties escaping [relational dimension]*. *Student-85 V1*

*I quickly went to catch the Mouse to change roles [cognitive dimension] and to be able to rest [organic dimension]*. *Student-90 V1*

*My strategy when I was a Cat was to go quickly after the Mouse [cognitive and organic dimension], be faster than him [organic dimension], and catch him [relational dimension] before he joined two other companions [relational dimension]*. *Student-73 V1*

This strategy can be an antidote to the emotional discomfort of being in the Cat role for a prolonged period of time.

*The most intense emotion I had with the role of the cat was sadness because one of the times I was a cat, I had a hard time catching the mouse [relational dimension] and being able to save myself from this role [relational dimension] and chasing it for a long time [relational dimension]*. *Student 14 V1*

Emotions, decisions and relationships merge in multidimensional subjective time. While referring to strategy, the following comment could also be a testimony to emotional experience.

*My time strategy in the Cat role was to go quickly for the Mouse [cognitive and organic dimension], as I did not like being in the Cat role [emotional dimension], and I wanted to get rid of it quickly*. *Student-23 V1*

Although there are no statistically significant differences, the data show that most negative emotions tend to be more intense in V1 than in V2.

Sometimes, the frustration of pursuing without achieving the goal causes anger to be the most intense emotion among the negative emotions (V1:M = 2.2; V2:M = 2.1).

*In this role, I felt angry when I tried to chase the Mice, and I noticed my proposed strategy [cognitive dimension] didn’t work*. *Student 78 V1*

*Often, the Mouse wants to ensure that the Cat does not catch him and quickly changes roles*. *Such decisions can generate comfort and discomfort at the same time*.

*In the role of the Cat, my intense emotions were joy and fear because the role changes were made very quickly [cognitive dimension]*. *Student 32 V1*

Despite the pressure that the Cat role assumes, as has been shown in other studies [16], "succeed actions (role changing) are not inscribed in a successive point system that is recorded in the game. At the end of the game, there is no quantified summary of marking actions. This feature, which de-dramatises ineffective actions and situations of failure, is a major factor in the range of emotions experienced by the participants" pp. 49–50.

*I like to be part of the action [organic dimension], but I’m always a bit afraid that I’m stuck doing the same role [cognitive dimension], and I may not be able to catch [relational dimension]*. *Student 31 V1*

Once again, strategic time is also emotional, relational and organic time.

#### 4.3.2. Version 2. The change of role from Cat to Mouse. The enemy Mouse becomes a friendly Mouse

In the second version, the rule change modifies the role change system, drastically transforming the Cat’s temporal plot. Players must unlearn the temporal patterns established in the first version to avoid falling into the trap of decisional confusion.The players’ bodies are signs to be deciphered. Each motor action carries the union of a signifier (observable motor behaviour) and a signified (underlying tactical project) [16]. In V2, the exact behaviour of the players (provoking the exit of a Pitcher player) causes an opposite signification and reaction. The Cat (G_1_) that was a predator a moment ago becomes prey (R_2_) since the Pitcher that leaves becomes a Cat (G_2_) [16].

The findings show that Cats in Version 2 take more time before capturing the Mouse and decrease the number of people (Mice) they interact with. The results of the classification trees show that the Chasing Cat only interacts motorically with 1 Mouse (LR = 1 interaction). Moreover, in V2, the Chasing Cat will spend more time in sedentary and light efforts.

All this justifies that the subjective perception of time in V2 differs from V1, where players perceive that the role changes from Cat to Mouse more quickly. The change of the rule entails changes in strategies and also in emotional experience.

*The most intense emotion I felt with the role of the Cat was joy because, in this modality, when the Mouse got hooked on a Pitcher [cognitive and relational dimension], I acquired this role [cognitive dimension]. I could save myself [relational dimension] and not be in the role of Cat for a long time [cognitive dimension]*. *Student 15 V2*

In V2, players learn there is no need to rush into chasing the Mouse, as the Mouse triggers the role change in a third person (a Pitcher going out as a Cat). On many occasions, the Mouse himself/herself will do the job by teaming up with a Pitcher. In these circumstances, the players feel well-being.

*I was agitated because what I was doing was chasing a mouse [relational dimension], but I knew that, at that moment, right after, I could be caught [relational dimension]. This fact created an uncertainty [cognitive dimension] that I liked*. *Student 16 V2*

A close reading of the game’s dynamics (analysis of the internal logic and its relationship to the findings)identifies a variety of Cat strategies at two different times in the game sequence.

*Moment 1*. *Strategy at the beginning of the sequence*. This moment is when the first Cat (G1) opposes the fleeing Mouse (R1). In this case, taking some time before acting is a good option. The data show that this is the predominant strategy. The players carry out their strategy with different internal meanings. The subjective time is decisional and also a relational time.

*In this modality, you cannot go for the Mouse [cognitive dimension] because if you hook into a Pitcher [cognitive and relational dimension], the role will change [cognitive dimension], and you will be caught [relational dimension]*. *Student-4 V2*

*My temporary strategy, being a Cat, was to wait for the change [cognitive dimension] to take place before running away [relational dimension] because the Pitcher was turning into a Mouse [cognitive dimension]*. *Student-48 V2*

To a lesser extent, some people employ a mixed strategy; the decision is bold but also considered. Each player must guess at the probabilities of success, appreciate their decisional options and act by taking the risk they think is appropriate [16].

*It was to go quickly [cognitive and organic dimension] for the Mouse [relational dimension], but always carefully when approaching a Pitcher [relational dimension] because I was going from being a Cat to being a Mouse at that moment [cognitive dimension]*. *Student-48 V2*

*I have varied the strategy according to the situation. Sometimes, I had to get out faster [cognitive and organic dimension] because I had the Mouse within reach [relational dimension]. Still, sometimes, because I knew that the Mouse was going to join the next pitcher [cognitive and relational dimension], I had to backtrack or improvise [cognitive dimension] so that I could save myself [relational dimension] when I changed roles [cognitive dimension]*. *Student-53 V2*

*Moment 2*. Just when a Pitcher transforms into a Cat. In V2, the role change system places the Cat in a second moment of great strategic interest, just when he transforms his role from Pitcher to Cat (G2). At that moment, the hesitation of the previous Cat (G1), now transformed into a Mouse (R2), must be taken advantage of. Depending on the opponent, this is an excellent moment to chase R2 quickly.

*As the cat role, my temporal strategy has been to go quickly [cognitive and organic dimension] after the mouse [relational dimension] because every time someone latched on to my partner [relational dimension], I already knew it was the Cat [cognitive dimension]. Therefore, it was straightforward for me to catch him off guard [relational dimension]*. *Student-2 V2*

*I tried to quickly go after the mouse [cognitive and relational dimension]*, *i.e. as soon as I was caught [relational dimension], promptly go after the mouse [cognitive and relational dimension] to change roles and run away [cognitive and relational dimension] Student-21 V2*

Players who internalise this new temporal order have correctly interpreted the game’s internal logic and have been able to adapt their strategic motor conduct to the different temporal fragments of each sequence of motor actions.

V2 fosters confusing decisions that often cause shared laughter and emotional complicity that triggers well-being in a relaxed atmosphere. Playtime is internalised as affective, relational and cognitive time.

*The most intense emotion was joy, as I had a good time catching people [relational dimension], as we confused the concepts of cat and mouse [cognitive dimension], and we had a good time with our companions [relational dimension]*. *Student 110 V2*

In other cases, people who have not appropriated the new role-changing network feel emotional discomfort because of this cognitive dissonance.

*When I was a cat, I felt anger with great intensity because I didn’t understand the game, I felt confused, I was angry that I didn’t understand it, I got confused, I lost [cognitive dimension]* … *Student 6 V2*

*The most intense emotion in the cat role was rejection and anger because, at first, I didn’t understand how the game worked [cognitive dimension]*. *Student 60 V2*

### 4.4. The temporary plot in the role of Mouse, friend or enemy in the game?

The data obtained show that players spend 10% of the game playing the subrole of Mouse-Sniffer (Mx). The Mouse is another of the main actors in this playful ritual [6, 13]. Modifying the role change system also affects the role changes of the Mouse. However, statistical tests recorded no significant differences in Mouse’s strategies in both modalities.

#### 4.4.1 The Mouse and the Cat, declared enemies by the internal logic

In both versions, the motor interaction with the Cat does not change; both are adversaries united by oppositional motor interactions. In V1 and V2, Mouse must prevent the Cat from capturing him to avoid swapping roles with the Cat. However, the system of role changes gives it a different prominence in each version. In Fig 2, the network offers a connected network, meaning all players can go through all roles between several sequences. However, in V1, the network shows a substantial difference in connectivity property. Internal logic demands that in V1, all players must cycle through the Mouse role to change roles. This feature confirms the crucial importance of this role [16]. In contrast, in V2, the change from Pitcher to Cat does not involve going through the role of Mouse.

The study of T-patterns reveals that it is the subrole that generates the highest number of multidimensional strategic chains (22) and also the most increased complexity (number of linked chains) (see Table 1). This strategic versatility may explain the emotional well-being that the role of the Mouse elicits.

Joy because, as a Mouse, you can use many strategies, types of moves and feints [cognitive dimension]. Student 3 V2

The Mouse is the starring role that takes all the participants’ attention.

*I like the role of the Mouse very much because I like people [relational dimension] to see what I can do [cognitive dimension]*. *Student 30 V1*

It seems justified that this role reports the most intense values of joy. The emotional scores are similar in both versions (V1: 5.3 out of 7; V2: 5.2 out of 7). In the face of such openness in decision-making, feeling empowered triggers well-being. Players like the feeling of being in control of the game and being able to decide what to do.

*Being Mouse was the role I enjoyed the most because I could change roles whenever I wanted [cognitive dimension]. So, I played with the Cat [relational dimension], and when I was in danger, I would join a Pitcher [cognitive and relational dimension]. However, when I was not in danger, I exposed the Cat to danger [relational and cognitive dimension]*. *Student 3 V2*

It seems justified that this role reports the most intense version 1; many players in the Mouse role want to ensure that the Cat does not catch them and choose an effective and straightforward strategy of joining a nearby Pitcher to save themselves.

*My temporary strategy in the role of Mouse has been to go quickly to the chosen Pitcher [cognitive and organic dimension] as I wanted to avoid risking or moving into the role of Cat (cognitive dimension]*. *Student 35 V1*

The possibility of being able to change roles by being caught by the Cat gives rise to negative emotions such as fear. This emotion is more intense in V1 because the Cat is in that role longer, and the mice feel the pressure.

*The role of the Mouse was scary because I didn’t know if I would be caught [relational dimension] or if I would have time to catch a Pitcher at the necessary speed [organic dimension]*. *Student 60 V1*

*Mode 2 also activates fearful emotional states in the Mouse when it flees*.

*Some players are looking for a simple strategy to avoid making a wrong decision in the face of the decisional complexity of the game*.

*My temporary strategy in terms of Mouse was to go quickly to the chosen Pitcher [cognitive dimension] as the game was more confusing, and I didn’t want to complicate it [cognitive dimension]*. *Student 43 V2*

In this version 2, the possibility of changing roles also generates fear.

*I felt a little afraid, but very little because I was trying not to get caught [relational dimension]*. *Student 4 V2*

*In Mouse’s role, the most intense emotion I had was Fear because I had the tension of having a Cat chasing me*. *Student 45 V2*

At the same time, the strategy can have an energetic motive. The Mouse can decide the physical effort to be made, a circumstance that makes some players feel good about playing this role.

*I was happy to take part in the game in the role of Mouse [cognitive dimension], as it is not the most tiring of all [organic dimension] because you don’t have to chase, but you are chased [relational dimension]*. *Student 114 V1*

The data show that in the second version (V2), there are more changes from Mouse to Pitcher than in V1. In addition, in V2, the Mouse spends more time performing actions with greater intensity (Vm2) and with very few steps. We interpret that far from having to worry about running away, it often benefits from the time taken by the Cat-Pursuer to move towards the Pitcher of interest. Furthermore, the classification tree groups all of the Mouse’s subroles (fleeing, joining a Marmoset or being captured) into a single node, which means that the Mouse tends to generate a very low number of motor interactions with other people.

*The temporary strategy in this case was to go quickly for the chosen Pitcher without overthinking [cognitive and organic dimension] as he was a bit tired [organic dimension], and the usual decision was to go for the nearest Pitcher [cognitive dimension]*. *Student 40 V2*

The game of the Singers activates socio-motor empathy [2]. This concept, which incorporates motor praxeology, corresponds to:

"A process by which the interacting person tries to grasp the point of view of the other participants. Empathic behaviours include cognitive aspects (appreciation of speeds, displacements and strategic operations, semio-motor decoding, etc.) and affective aspects (perception of emotions, fears and aggressiveness, desire to succeed at all costs …). This process requires the abandonment of one’s point of view and the adoption of another centre of perspective", p. 188 [13].

*It was fun [emotional dimension] to watch the Cat try to catch you [relational dimension]* *and to have to decide which Pitcher you hooked onto [cognitive and relational dimension] to make it easier for him to catch the Cat [relational dimension] Student 34 V2*

The Mouse interprets the rival, assesses its possibilities and plays a leading role in a strategy where relationship and emotion confirm the socio-motor empathy that activates this role.

*I preferred to entertain myself for a while to play [emotional dimension] and to have a good time with the Cat [emotional and relational dimension]. So, I took more time before changing roles [cognitive dimension]*. *Student 12 V2*

Since there is no final score, it is easier for the players in the Mouse role to engage in empathic motor behaviour with the other participants who could be more active in the role of Piggy Bank.

*Regarding strategy as a Mouse, I have taken my time before going to a pitcher [cognitive dimension], staying short because otherwise, my colleagues would not play [relational dimension]*. *Student 29 V2*

The evidence from this study reveals that V2 leads to more Mouse-to-Cat switches than V1. One possible explanation for this is due to the confusion caused by the alteration of the role change system. As previously indicated, the confusion makes catching a Mouse easier for the Cat. So, for some Mice, there is insecurity or fear of not succeeding in escaping from a simple and effective strategy.

*I didn’t spend time playing the Cat [relational dimension]; it’s a matter of confidence or not feeling confident [emotional dimension], trying to escape [relational dimension]*. *Student 87 V2*

Each game imposes a universe of actions circumscribed in its internal logic. Initially, the player’s actions are indecisive, but the players gradually adapt to these constraints until they feel integrated into the internal logic; they have acquired a sense of the game (just as linguists speak of a feeling for the language). p.67 [13].

In both versions, decoding the temporal plot facilitates to sense the game and appropriately build a more complex strategy.

*At first, I would run to the nearest Pitcher to get out of that role [cognitive dimension]. Then I saw that the participation time was very short, and I took a little more time to play with the Cat [relational dimension]; I placed myself behind a Pitcher [relational dimension]*. *Student 18 V1*

On the other hand, as long as the players have not appropriated that sense of the game, negative emotions are likely to be very present.

*When I was Mouse, I was angry that when I changed roles [cognitive dimension], at the beginning, I was not very clear about the rules or the dynamics [cognitive dimension]. I got caught unconsciously [relational dimension] or confused with the roles [cognitive dimension]*. *Student 40 V2*

Modifying the rule in a game with complex relationships increases the difficulty of internalising the meaning of the game’s plot. It seems reasonable that in V2, the Anger tends to reach more intense values than in V1.

*When I was Mouse, I would last up to 1 second in this role as I would get confused and experience rage*. *Student 6 V2*

In both modalities, the Mouse is responsible for generating ambivalent or paradoxical motor relations with the other participants. Thanks to its intervention, up to four people can be involved. The binary relationship between Cat and Mouse becomes ternary (Cat-Mouse-Mouse-Mouse joining) or even quaternary (Cat-Mouse-Mouse-Mouse joining and Mouse leaving) p. 48 [16].

In these circumstances, it is difficult to know whether the Mouse is a friend or foe of the Cat and of the Pitchers.

#### 4.4.2. V1. The Mouse (R_1_) enemy of another Mouse (R_2_) and friend of the Cat (G1) in V1

In version 1, the Mouse (R_1_) can harm the new Mouse (R_2_) and therefore favour the Cat (G_1_). The testimonies on strategies and emotions confirm this first possibility of relational ambivalence.

Comment on the strategy. *I played with the Cat [relational dimension]*, *standing behind the pitchers so that I could leave less vision [cognitive dimension]*. *When the Cat was close to a pitcher*, *I would hold on to one of them [relational and cognitive dimension]*, *and I would make it easier for the Cat to catch it [relational dimension] Student 78 V1*

Comment on emotions. *The role of the Mouse is a lot of fun; you can do a lot of feints depending on your style of play*. *In addition*, *the Mouse can either cooperate with the other teammates or can "betray" them and grab a quick Pitcher so that the new Mouse is caught by the Cat*, *who is already prepared near the corresponding Pitcher to catch him/her*. *Student 3 V1*

Comment on the strategy. *I have been in situations where I have been a bit superior and have made the Cat dizzy [cognitive and relational dimension]*. *The strategy of standing behind a Pitcher [cognitive dimension] and annoying the Pitcher because the Cat would be close by [relational dimension] comes into play*. *Student 39 V1*

In V1, sometimes the Mouse observes that he/she has just harmed a person (Pitcher) that he did not want to break, which can make him/her feel bad.

*In this case, anger because, as a Mouse, you are the one being persecuted [relational dimension], and many times you were caught [relational dimension], and the strategy you proposed to join a Pitcher was not the right one [cognitive dimension], because the partner who was a Mouse was caught [relational dimension]*. *Student 79 V1*

#### 4.4.3. V2. The Mouse (R1) friend of the second Cat (G_2_) and enemy of the first Cat (G_1_)

In version 2, the Mouse (R_1_) switching to the role of the Pitcher causes a double valence motor relation: it generates a negative motor interaction on a Pitcher by giving it the unfavourable role of a Cat (G_2_) and a positive motor interaction on the former Cat (G_1_) as it makes it switch to the positive role of Mouse (R_2_).

*I used the strategy of changing trajectory so that I could trick the Cat [cognitive dimension] and help my fellow Pitchers [relational dimension] so that they could catch it [relational dimension] when I changed roles [cognitive dimension]*. *Student 95 V2*

*In the role of the Mouse, my strategy was to take my time because the moment I approached a Pitcher [cognitive dimension] if the Cat was around [relational dimension], it would cause another one to come out as a Cat [cognitive dimension] and catch it [relational dimension]. So, I thought that if I could make the Cat tired [organic dimension], he/she would get caught very soon [relational and cognitive dimension]*. *Student 10 V2*

*When the Cat was close to the pitcher, I would hook myself to a Pitcher [relational and cognitive dimension] so that the new Cat could catch the new Mouse [relational dimension], and he/she would stay in the centre of the game*. *Student 43 V2*

The evidence confirms the power of the game’s internal logic in the praxical consequences it generates in the actors. The motor communication network is ambivalent due to the motor interaction that the Mouse exerts on the role change of the Pitchers. The Mouse is responsible for the person on the other side of the Pitcher coming out in a weak role (Mouse, R2 in V1) or a vital role (Cat, G2 in V2). [2, 7, 33].

The Mouse, which can be considered a weak role of the Cat in V1, intervenes in V2 as a strong role, as it can turn the Cat into a Mouse. At the same time, in both versions, the Mouse plays a strong role over the Pitchers, as it can decide who will change and remain in the same role. This explains why the study of T-Patterns establishes that the Mouse-Persecuted (MX) role is the one that gives rise to the most significant number of complex temporal patterns (22, in all six levels of complexity). That is to say; the Mouse is the origin of multidimensional strategy links that can have up to 6 chainings of other patterns consecutively.

## 5. Conclusions

The two studies that constitute this article provide interesting findings for interpreting the complex web of the social construction of playtime in an original traditional sports game. We highlight the following contributions.

### 1. Motor praxiology

The evidence confirms the solidity of the epistemological bases offered by motor praxiology. Revealing the game’s temporal plot implies interpreting the game’s internal laws (its internal logic, its role-change system). This step makes it possible to understand that the underlying game structure (system) governs the motor behaviours temporality and the motor conduct of the participants (actor). The internal logic dictates motor behavior and its meaningful organization, specifically, motor conduct [13, 28].

### 2. The methodology used

Mixed methods have facilitated data collection and analysis of the integrated results to reveal the phenomenon studied.

- The use of mixed methods has combined a diversity of strategies for data collection, from external observation (observational analysis of roles and subroles, the number of interactions; objective analysis of physical effort) with the internal approach (content analysis of the testimonies on strategies and emotions).
- The two studies have combined a variety of instruments (observation sheets of subroles and motor interactions, recording of accelerometer numbers, temporal databases, with second-by-second recordings, global databases, with the perception and analysis of the physical effort) with the internal analysis (content analysis of the testimonies on strategies and emotions).
- The two studies have combined a variety of instruments (observation sheets of subroles and motor interactions, recording of accelerometer numbers, temporal databases, with second-by-second records, global databases, with global perception of the game).
- The research has combined different statistical techniques (cross tables, classification trees, T-Patterns), which have led to the integrated interpretation of all the data.
- The methodology used may be of great interest to be replicated in other studies, not only in the context of quality physical education but also in confirming the pedagogical benefits of the traditional sports game.

### 3. The Elbow Tag game. An original role change system

- The Pitcher game contains an original internal logic articulated by the transition between the roles of Cat, Mouse and Pitcher, which is very different in the two versions.
- It is found that a simple change in a rule can cause a profound change in the internal dynamics of the game, significantly if it substantially modifies the system of changing roles.
- The playful plot, which lasts eight minutes in each game mode, unfolds a variety of cyclical sequences that give rise to the ternary transition between the roles of Cat, Mouse and Pitcher. Social time is cyclical and unique in both versions.
- Understanding the system of role changes is critical to deciphering the temporal uniqueness of the Pitcher game.

### 4. Motor behaviour. The first level of analysis of the temporal plot

- To interpret the socialisation process of playtime, it is necessary to go beyond the external units of measurement of physics (objective physical time). Each person adapts in a very particular way to the temporal logic of play. External observation confirms the multidimensional nature of subjective time. The game originates cyclical temporal sequences of motor behaviours integrated by multifaceted strategies. These chains activate in a unitary manner the cognitive (roles and subroles), relational (motor interactions), and organic (physical effort and steps) planes; this finding has also been observed in other games (e.g., [4, 5, 16, 33]).
- The two versions of the Elbow Tag game have a system of unequal role changes. The internal logic causes different effects on the motor behaviours of the two versions. Identifying T-Patterns through traditional games confirms their efficacy and scientific interest, as seen in previous studies (e.g., [18]).
- To identify multidimensional strategic linkage chains (decisional, relational and organic), observing the motor behaviours used to solve a motor situation and finding out when they occur is essential. This approach implies an in-depth analysis involving a process of temporal discrimination rather than simply juxtaposing motor behaviours. Sixty-seven temporal patterns have been identified, more complex in number of chaining in V2 (reaching up to 5 T-Patterns, as opposed to V1, which reaches a maximum of 3 T-Patterns). The second modality dedicates more time to high and moderate ranges of relationships (number of people with whom each role interacts) and also to vigorous efforts.

### 5. Motor conducts. The second level of analysis of the time frame

- The second study allows us to go deeper into a second level of investigation and interpretation. The Motor Behaviours, captured from the outside, show limits to understanding the subjective meaning that people give to this temporal plot of the game; for this reason, it is necessary to the understanding of Motor Conduct, that is to say, to the subjective meaning that people give to the temporal plot of the game. The players’ accounts express this internal meaning, which is invisible to an external gaze.
- The temporal plot is forged from the deep structure of the international logic of the game [5], that is, from the network of role changes, which is different in both modalities.
- The Pitcher Role shows that it plays a crucial role in internalizing the temporal plot of the game. Passive from a motor point of view, but very active in the attentive observation of the game and the experience of fleeting and changing emotions of well-being (due to the group environment). This Role also causes discomfort due to motor passivity, fear of changing roles, and not correctly interpreting the game’s internal logic). The subjective time of the Pitcher is also a cognitive, emotional and relational time.
- The Cat Role is one of the two main actors in this playful ritual. Their strategies vary between the two versions; in V1, the Cat tends to go quickly for the Mouse, whereas in V2, they wait longer before capturing it. Two key moments can be distinguished: when starting the sequence as a Cat (in V1 and V2) or when a player moves from the Role of Piglet to a Cat (in V2). In this Role, emotional well-being comes from feeling powerful, from having unravelled the temporal plot of the game, from the temporal pressure received and from the confusion of the companions. The Cat also expresses discomfort at the frustration of not capturing a Mouse (in V1) or at the bewilderment in interpreting the temporal laws of the game (in V2). The subjective time of the Cat is also a cognitive, emotional, organic and relational time.
- The Role of the Mouse is the leading actor in the playful plot, which is why it is the Role that originates the greatest number of T-Patterns or multidimensional strategic chains in the game. The strategies are similar in V1 and V2. They are built from the search for efficiency, effort control, empathy for others, deciphering the game’s internal logic, decoding the opponent’s signs, and feeling good with the Cat and insecurity. This Role is a carrier of ambivalence in the game and can act as an enemy of the next Mouse and a friend of the Cat, as an enemy of the Pitcher and a friend of the Cat. In the Role of the Mouse, the players feel well-being for taking the initiative, taking the centre of attention, feeling empowered, the effort to flee, and the paradoxical relationships it provokes. They also experience discomfort with the change of Role to Cat for not understanding the logic of the temporal game. The Mouse can participate in a relational ambivalence and also an emotional ambivalence. The subjective time of the Mouse is also a cognitive, emotional, organic and relational time.

### 6. Transfer for quality physical education

- Understanding the temporal process in the development of motor behaviour during the game brings a new look for new physical education, which is traditionally too much entertained in physical time (calculated with external temporal units).
- This new perspective of subjective time is aligned with the education of Motor Conduct. The teacher identifies the motor behaviours (with an astute external observation) and, through the students’ testimonies, recognizes the meaning they have given to their motor behaviours. It is then, from an educational project, that he/she tries to personalize the optimization of Motor Conduct. In this case, the challenge would be to promote the well-being of all students by learning to unveil internal logic and to adjust their Motor Conduct to the time frame required by each game. The multidimensional vision of subjective time will sometimes lead to the education of relational, decisional, emotional or organic units.
- Motor Conducts are a mirror reflecting the civilizing process of time, which involves disciplining, controlling and moderating the impulses of the personal biological clock (individual self-action, psychogenetic) to adjust them to physical time (objective) and social time (relational clock), according to the rules and codes of respectful behaviour established by society or community (external social coercion, sociogenetic) [1].
- Thanks to sharing an original, playful time originated by a framework of cyclical relational exchanges, the pupils live a state of affective transition, inscribed in a liminal time [13] full of symbols that generate deep affective meanings [9, 13, 19]. Each game is a source of deep temporal sensations, which the pupils share through a singular civilizing process that helps to generate a state of belonging to their playful community.
- This experience it has been a useful task of this new physical education: to help build our students’ social time. Endeavoring to build, alongside our students, a conception of time is to enhance the quality of physical education.
- Recommendations for Educators and Policymakers: In light of the findings from this study, it is evident that educators should incorporate a deeper understand-ing of subjective time and its impact on physical education into their pedagogical practices. It is recommended that teacher training programmes include strategies to identify and analyse not only physical time but also the subjective time experienced by students in traditional sports games. Additionally, policymakers should consider integrating these approaches into physical education curricula, ensuring that educational practices promote not only physical competence but also the emotional, relational, and decisional development of students. This aligns with the need for a more holistic physical education that prepares students to engage effectively and mean-ingfully in their communities.
- Generalisation of Findings to Other Traditional Sports Games (TSGs) and Cultural Contexts: The results obtained in this study on Elbow Tag can be extrapolated to other TSGs with similar characteristics, such as the absence of a final score or a direct competition for victory. These games, like Elbow Tag, may offer unique tem-poral and affective experiences that enhance social cohesion and a sense of belonging within educational communities. Moreover, replicating this approach in diverse cul-tural contexts, where TSGs may have different meanings and values, would be valuable. Adapting the findings of this study to other cultures will allow for exploration of how different conceptions of time and social structures affect the way students expe-rience and benefit from TSGs, thereby enriching global educational practices.

Finally, as a limitation to further research, exploring the personalized vision of the temporal patterns of behaviour and the meaning of the Motor Conduct of specific players is suggested. This approach would allow a deeper understanding of the subjective meaning, proposing complementary techniques such as group interviews. Likewise, it would be interesting to deepen the temporal plot’s study from a gender perspective or to carry out this same study with primary or secondary school students.

Another relevant limitation for future research pertains to the cultural and sporting context of the participants in this study. The students from the Faculty of Physical Activity and Sport Sciences, who constituted our sample, have a well-defined sports background, with the majority engaging in regular sports activities. This specific participant profile may influence their perception and behaviour within traditional games such as Elbow Tag, which features a temporality distinct from modern competitive sports, where there is no final score or competition for victory or defeat. Their familiarity with sports characterised by linear and competitive temporal structures could have shaped their experience and interpretation of the roles in the game.

In this regard, it would be valuable to replicate this study with other groups of university students whose studies are not related to physical activity and sports, where the proportion of regular sports practitioners is lower. Such a comparison would explore how the absence of a defined sports orientation might affect the experience of temporality and interactions in traditional games. Furthermore, a deeper analysis from a gender perspective and in different educational contexts, such as primary or secondary school students, could provide a richer understanding of how these factors influence the perception and meaning of traditional games across various cultural contexts.

These suggestions would not only contribute to a broader generalisation of the findings but also address potential cultural biases inherent in the specific context of our sample, thereby strengthening the credibility and applicability of our study’s conclusions.

## Supporting information

S1 Appendix (DOCX)

## References

[1] Elias N. Sobre el tiempo. México: Fondo de Cultura Económica; 1984.

[2] Piaget J. Le développement de la notion de temps chez l’enfant. Paris: PUF; 1981.

[3] Marijuá PC, Montero-Marín J, Navarro J, García-Campayo J, del Moral R. The “sociotype” construct: Gauging the structure and dynamics of human sociality. 2017 [cited 13 May 2024]. doi: 10.1371/journal.pone.0189568 29240816 PMC5730176

[4] Lavega-Burgués P, Alcaraz-Muñoz V, Mallén-Lacambra C, Pic M. Roles, relationships, and motor aggressions: Keys to unveiling the emotions of a traditional sporting game. Front Psychol. 2023;14. doi: 10.3389/fpsyg.2023.1127602 36798892 PMC9926965

[5] Martín-Martínez D, Lavega-Burgués P, Salas-Santandreu C, Duran-Delgado C, Prat Q, Damian-Silva S, et al. Relationships, Decisions, and Physical Effort in the Marro Traditional Sporting Game: A Multimodal Approach. Int J Environ Res Public Health. 2021;18: 10832. doi: 10.3390/ijerph182010832 34682577 PMC8535934

[6] Huizinga J. Homo Ludens: el elemento lúdico de la cultura. Madrid: Alianza; 1972.

[7] Turner VW. From Ritual to Theatre: The human seriousness of play. Michigan: Performing Arts Journal Publications; 1982.

[8] Goffman E. Frame Analysis. Los marcos de la experiencia. Madrid: Centro de Investigaciones Sociológicas; 2007.

[9] Costes A, March-Llanes J, Muñoz-Arroyave V, Damian-Silva S, Luchoro-Parrilla R, Salas-Santandreu C, et al. Traditional Sporting Games as Emotional Communities: The Case of Alcover and Moll’s Catalan–Valencian–Balearic Dictionary. Front Psychol. 2021;11: 1–9. doi: 10.3389/fpsyg.2020.582783 33536964 PMC7848262

[10] Rosenwein BH. Generations of feeling: A history of emotions, 600–1700. Cambridge: Cambridge University Press; 2016.

[11] Smith P. The Balinese Cockfight Decoded: Reflections on Geertz and Structuralism. Interpreting Clifford Geertz. 2011; 17–32. doi: 10.1057/9780230118980_3

[12] Csikszentmihalyi M. Flow: The Psychology of Optimal Experience. 1990.

[13] Parlebas P. Juegos, Deporte y Sociedad. Léxico de praxiología motriz. Editorial Paidotribo; 2001.

[14] Suits B. The grasshopper: Games, life and utopia. Toronto: University of Toronto Press; 1978.

[15] Etxebeste J, Del Barrio S, Urdangarin C, Usabiaga O, Oiarbide A. Ganar, perder o no competir: la construcción temporal de las emociones en los juegos deportivos. Educatio Siglo XXI. 2014;32: 33–48. doi: 10.6018/J/194051

[16] Parlebas P. Jeux traditionnels et dynamique relationnelle. L. Collard. Sport & bien-être relationnel Un autre aspect de la santé. L. Collard. Paris: Chiron; 2012. pp. 41–86.

[17] Muñoz-Arroyave V, Lavega-Burgués P, Costes Rodríguez A, Prat Q, Serna J. University students’ affective experiences while playing: A qualitative perspective. Current Psychology. 2021. pp. 1133–1143. doi: 10.1007/s12144-018-0028-z

[18] Muñoz-Arroyave V, Pic M, Luchoro-Parrilla R, Serna J, Salas-Santandreu C, Damian-Silva S, et al. Promoting interpersonal relationships through elbow tag, a traditional sporting game. A multidimensional approach. Sustainability (Switzerland). 2021;13. doi: 10.3390/su13147887

[19] Parlebas P. La aventura praxiológica. Ciencia, acción y educación física. Sevilla: Consejería de Turismo y Deporte; 2017.

[20] Besharati Holasoo M, Naderinasab M, Ramezaninejad R. Modeling the National Pattern of Sports Development in Iranian Families. AI and Tech in Behavioral and Social Sciences. 2024;2: 35–45. doi: 10.61838/kman.aitech.2.1.5

[21] Rahmani N, Naderi Nasab M, Taheri M, Biniaz SA. Exploring the Future of the Sports Industry Through an Economic Lens in 2031. International Journal of Innovation Management and Organizational Behavior. 2024;4: 170–179. doi: 10.61838/kman.ijimob.4.1.20

[22] Anguera MT, Blanco-Villaseñor A, Losada JL, Portell M. Pautas para elaborar trabajos que utilizan la metodología observacional. Anuario de Psicología. 2018;48: 9–17. doi: 10.1016/J.ANPSIC.2018.02.001

[23] Teddlie C, Tashakkori A. Overview of contemporary issues in mixed methods research. 2nd ed. Sage handbook of mixed methods in social & behavioral research (. 2nd ed. London: Sage; 2010. pp. 1–44.

[24] Ato M, López JJ, Benavente A. Un sistema de clasificación de los diseños de investigación en psicología. Anales de Psicologia. 2013;29. doi: 10.6018/analesps.29.3.178511

[25] Anguera MT, Blanco-Villaseñor A, Jonsson GK, Losada JL, Portell M. Editorial: Best Practice Approaches for Mixed Methods Research in Psychological Science. Front Psychol. 2020;11. doi: 10.3389/fpsyg.2020.590131 33424707 PMC7793979

[26] Argilaga MTA, Villaseñor ÁB, Mendo AH, López JLL. Diseños Observacionales: Ajuste y aplicación en psicología del deporte. Cuadernos de Psicología del Deporte. 2011;11: 63–76. Available: https://revistas.um.es/cpd/article/view/133241

[27] Parlebas P. The Universals of Games and Sports. Front Psychol. 2020;11. doi: 10.3389/fpsyg.2020.593877 33192937 PMC7609522

[28] Krippendorff K. Metodología de análisis de contenido: Teoría y práctica. Barcelona: Paidós; 2002.

[29] Miles MB, Huberman AM. Qualitative data analysis. 2nd ed. Thousand Oaks: Sage; 1994.

[30] Taylor S, Bogdan R. Introducción a los métodos cualitativos de Investigación. Barcelona: Paidós; 2000.

[31] Levitt HM, Bamberg M, Creswell JW, Frost DM, Josselson R, Suárez-Orozco C. Journal article reporting standards for qualitative primary, qualitative meta-analytic, and mixed methods research in psychology: The APA Publications and Communications Board task force report. American Psychologist. 2018;73: 26–46. doi: 10.1037/amp0000151 29345485

[32] Casarrubea M, Davies C, Pierucci M, Colangeli R, Deidda G, Santangelo A, et al. The impact of chronic daily nicotine exposure and its overnight withdrawal on the structure of anxiety-related behaviors in rats: Role of the lateral habenula. Prog Neuropsychopharmacol Biol Psychiatry. 2021;105: 110131. doi: 10.1016/j.pnpbp.2020.110131 33039434

[33] Etxebeste J. À cloche-pied: Les jeux sportifs et la socialisation des enfants basques. London: Université Européene; 2012.

[34] González Monteagudo J. El paradigma interpretativo en la investigación social y educativa: nuevas respuestas para viejos interrogantes. Cuestiones pedagógicas. 2001;15: 227–246.

[35] Pic M, Jonsson GK. Professional boxing analysis with T-Patterns. Physiol Behav. 2021;232. doi: 10.1016/j.physbeh.2021.113329 33493543

[36] Magnusson MS. Discovering hidden time patterns in behavior: T-patterns and their detection. Behavior Research Methods, Instruments, & Computers. 2000;32: 93–110. doi: 10.3758/bf03200792 10758668

[37] Lavega-Burgués P, March-Llanes J, Moya-Higueras J. Validation of games and emotions scale (GES-II) to study emotional motor experiences. Revista de Psicologia del Deporte. 2017;27: 117–124.

[38] Lagardera F, Lavega P, Etxebeste J, Alonso JI. Metodología cualitativa en el estudio del juego tradicional [Qualitative Methodology in the Study of Traditional Games]. Apunts Educación Física y Deportes. 2018; 20–38. doi: 10.5672/APUNTS.2014-0983.ES.(2018/4).134.02

[39] Anguera MT, Blanco A. Registro y codificación en el comportamiento deportivo. Metodología. Buenos Aires: Efdeportes; 2003. pp. 6–34.

[40] Gomez MA, Rivas F, Connor JD, Leicht AS. Performance Differences of Temporal Parameters and Point Outcome between Elite Men’s and Women’s Badminton Players According to Match-Related Contexts. Int J Environ Res Public Health. 2019;16. doi: 10.3390/ijerph16214057 31652686 PMC6862575

[41] Cohen J. Statistical Power Analysis for the Behavioral Sciences. New York: Routledge; 2013. doi: 10.4324/9780203771587

