# Supplementary material for: The development of social sustainability through traditional sporting games. A temporal approach with the Elbow Tag
Source: PLoS One. 2024 Nov 25;19(11):e0312092. doi: 10.1371/journal.pone.0312092 (PMC11588213; doi:10.1371/journal.pone.0312092)
Supplement: S1 Appendix — (DOCX) [file pone.0312092.s001.docx]

**Appendix A**

**Table A6. Cyclic transition time between subroles in v1 and v2**

|  | | | Pitcher_Version_1_2 | | Total  (frequency) |
| --- | --- | --- | --- | --- | --- |
|  |  |  | 1 | 2 |  |
| Subroles | DC | Count | 42 | 29 | 71 |
|  |  | % of Total | 0.9% | 0.6% | 1.5% |
|  |  | Adjusted Residual | 1.7 | -1.7 |  |
|  | CF | Count | 44 | 43 | 87 |
|  |  | % of Total | 0.9% | 0.9% | 1.9% |
|  |  | Adjusted Residual | ,2 | -,2 |  |
|  | CT | Count | 269 | 248 | 517 |
|  |  | % of Total | 5.7% | 5.3% | 11.0% |
|  |  | Adjusted Residual | 1.3 | -1.3 |  |
|  | MC | Count | 83 | 89 | 172 |
|  |  | % of Total | 1.8% | 1.9% | 3.7% |
|  |  | Adjusted Residual | -,3 | ,3 |  |
|  | M.J. | Count | 44 | 31 | 75 |
|  |  | % of Total | 0.9% | 0.7% | 1.6% |
|  |  | Adjusted Residual | 1.6 | -1.6 |  |
|  | MX | Count | 229 | 249 | 478 |
|  |  | % of Total | 4.9% | 5.3% | 10.2% |
|  |  | Adjusted Residual | -.7 | .7 |  |
|  | PL | Count | 78 | 80 | 158 |
|  |  | % of Total | 1.7% | 1.7% | 3.4% |
|  |  | Adjusted Residual | ,0 | ,0 |  |
|  | POH | Count | 1528 | 1608 | 3136 |
|  |  | % of Total | 32.6% | 34.3% | 66.8% |
|  |  | Adjusted Residual | -1.2 | 1.2 |  |
| Total | | Count | 2317 | 2377 | 4694 |
|  |  | % of Total | 49.4% | 50.6% | 100.0% |

Note. CC Cat-Catcher; CF Conflict; CT Cat-Purser; MC Mouse-Eastener; MJ Mouse-Caught; MX Mouse-Pursued; POH Pitcher-Waiting.

| Chi-Square Tests | | | |
| --- | --- | --- | --- |
|  | value | df | Asymptotic Significance (2-sided) |
| Pearson Chi-Square | 7,845a | 7 | .346 |
| Likelihood Ratio | 7,869 | 7 | .344 |
| N of Valid Cases | 4694 |  |  |

Note. Zero cells (0.0%) have expected count less than 5. The minimum expected count is 35.05.

| Symmetric Measurements | | | |
| --- | --- | --- | --- |
|  | | value | Approximate Significance |
| Nominal by Nominal | Phi | .041 | .346 |
|  | Cramer's V | .041 | .346 |
| N of Valid Cases | | 4694 |  |

**Table A7.** **Comparison of the cyclic time of physical effort (vector magnitude Vm^2^) and steps in V1 and V2**

|  | | | Pitcher_Version_1_2 | | Total |
| --- | --- | --- | --- | --- | --- |
|  |  |  | 1 | 2 |  |
| *Vm^2^* | L | Count | 730 | 773 | 1503 |
|  |  | % of Total | 15.6% | 16.5% | 32.0% |
|  |  | Adjusted Residual | -.7 | .7 |  |
|  | M | Count | 340 | 346 | 686 |
|  |  | % of Total | 7.2% | 7.4% | 14.6% |
|  |  | Adjusted Residual | ,1 | -,1 |  |
|  | S | Count | 572 | 623 | 1195 |
|  |  | % of Total | 12.2% | 13.3% | 25.5% |
|  |  | Adjusted Residual | -1.2 | 1.2 |  |
|  | V | Count | 675 | 635 | 1310 |
|  |  | % of Total | 14.4% | 13.5% | 27.9% |
|  |  | Adjusted Residual | 1.8 | -1.8 |  |
| Total | | Count | 2317 | 2377 | 4694 |
|  |  | % of Total | 49.4% | 50.6% | 100.0% |

Note. Effort magnitude value (Vm^2^): Sedentary: S (0-2 CPS); Light L (<2-34CPS); Moderate M >34-100 CPS; Vigorous V (>100CPS).

| Chi-Square Tests | | | |
| --- | --- | --- | --- |
|  | value | df | Asymptotic Significance (2-sided) |
| Pearson Chi-Square | 3,914a | 3 | .271 |
| Likelihood Ratio | 3,915 | 3 | .271 |
| N of Valid Cases | 4694 |  |  |

Note. to. 0 cells (0.0%) have expected count less than 5. The minimum expected count is 338.62.

| Symmetric Measurements | | | |
| --- | --- | --- | --- |
|  | | value | Approximate Significance |
| Nominal by Nominal | Phi | .029 | .271 |
|  | Cramer's V | .029 | .271 |
| N of Valid Cases | | 4694 |  |

**Table A8. The cyclic time of transition between roles in V1 and V2**

|  | | | Version | | Total |
| --- | --- | --- | --- | --- | --- |
|  |  |  | 1 | 2 |  |
| Transition | DC | Count | 299 | 194 | 493 |
|  |  | Expected Count | 243.3 | 249.7 | 493.0 |
|  |  | Adjusted Residual | 5.3 | -5.3 |  |
|  | CM | Count | 47 | 3. 4 | 81 |
|  |  | Expected Count | 40.0 | 41.0 | 81.0 |
|  |  | Adjusted Residual | 1.6 | -1.6 |  |
|  | MC | Count | 50 | 121 | 171 |
|  |  | Expected Count | 84.4 | 86.6 | 171.0 |
|  |  | Adjusted Residual | -5.4 | 5.4 |  |
|  | MM | Count | 227 | 245 | 472 |
|  |  | Expected Count | 233.0 | 239.0 | 472.0 |
|  |  | Adjusted Residual | -.6 | .6 |  |
|  | M.P. | Count | 79 | 23 | 81 |
|  |  | Expected Count | 40.0 | 41.0 | 81.0 |
|  |  | Adjusted Residual | 8.7 | -8.7 |  |
|  | PC | Count | 0 | 85 | 85 |
|  |  | Expected Count | 4.4 | 78.6 | 83 |
|  |  | Adjusted Residual | -9.7 | 9.7 |  |
|  | P.M | Count | 82 | 0 | 82 |
|  |  | Expected Count | 84 | 0 | 84 |
|  |  | Adjusted Residual | 9.8 | -9.8 |  |
|  | PP | Count | 1512 | 1671 | 3088 |
|  |  | Expected Count | 1524.1 | 1663.9 | 3088.0 |
|  |  | Adjusted Residual | -1.1 | 1.1 |  |
| Total | | Count | 2069 | 2427 | 4664 |
|  |  | Expected Count | 2306 | 2358 | 4664 |

Note. CC Cat-Catcher; CF Conflict; CT Cat-Purser; MC Mouse-Eastener; MJ Mouse-Caught; MX Mouse-Pursued; POH Pitcher-Waiting.

| Chi-Square Tests | | | |
| --- | --- | --- | --- |
|  | value | df | Asymptotic Significance (2-sided) |
| Pearson Chi-Square | 201,343a | 8 | p <.001 |
| Likelihood Ratio | 236,162 | 8 | p <.001 |
|  |  |  |  |
|  | | | |

| Symmetric Measurements | | | |
| --- | --- | --- | --- |
|  | | value | Approximate Significance |
| Nominal by Nominal | Phi | ,208 | p <.001 |
|  | Cramer's V | ,208 | p <.001 |
|  | |  |  |

**Table A9.** **Relational cyclic time in both versions V1 and V2**

|  | | | Pitcher_Version_1_2 | | Total |
| --- | --- | --- | --- | --- | --- |
|  |  |  | 1 | 2 |  |
| Range Relations | HR | Count | 106 | 50 | 156 |
|  |  | % of Total | 23% | 1.1% | 3.3% |
|  |  | Adjusted Residual | 4.7 | -4.7 |  |
|  | LR | Count | 1494 | 1668 | 3162 |
|  |  | % of Total | 31.8% | 35.5% | 67.4% |
|  |  | Adjusted Residual | -4.2 | 4.2 |  |
|  | MR | Count | 339 | 288 | 627 |
|  |  | % of Total | 7.2% | 6.1% | 13.4% |
|  |  | Adjusted Residual | 2.5 | -2.5 |  |
|  | SR | Count | 378 | 371 | 749 |
|  |  | % of Total | 8.1% | 7.9% | 16.0% |
|  |  | Adjusted Residual | .7 | -.7 |  |
| Total | | Count | 2317 | 2377 | 4694 |
|  |  | % of Total | 49.4% | 50.6% | 100.0% |

| Chi-Square Tests | | | |
| --- | --- | --- | --- |
|  | value | df | Asymptotic Significance (2-sided) |
| Pearson Chi-Square | 33,130a | 3 | *p*< .001 |
| Likelihood Ratio | 33,589 | 3 | *p*< .001 |

Note. LR (Low Relationships = 1); SR (Slight Relationships = 2); MR (Moderate LR (Low Relationships = 1); SR (Slight Relationships = 2); MR (Moderate Relationships = 3-4); HR (High Relationships =>5).

| Symmetric Measurements | | | |
| --- | --- | --- | --- |
|  | | value | Approximate Significance |
| Nominal by Nominal | Phi | .084 | *p*< .001 |
|  | Cramer's V | .084 | *p*< .001 |
| N of Valid Cases | | 4694 |  |

Note. to. 0 cells (0.0%) have expected count less than 5. The minimum expected count is 77.00.

**Table A10.** **Comparison of the cyclic time of physical effort (vector magnitude Vm^2^) in the roles of Cat and Mouse in V1 and V2**

| ROLE | | | | Pitcher | | Total |
| --- | --- | --- | --- | --- | --- | --- |
|  |  |  |  | 1 | 2 |  |
| C | Vm^2^ | L | Count | 21 | 39 | 60 |
|  |  |  | Expected Count | 31.6 | 28.4 | 60.0 |
|  |  |  | Adjusted Residual | -2.9 | 2.9 |  |
|  |  | M | Count | 54 | 57 | 111 |
|  |  |  | Expected Count | 58.4 | 52.6 | 111.0 |
|  |  |  | Adjusted Residual | -.9 | .9 |  |
|  |  | S | Count | 8 | 37 | Four. Five |
|  |  |  | Expected Count | 23.7 | 21.3 | 45.0 |
|  |  |  | Adjusted Residual | -4.8 | 4.8 |  |
|  |  | V | Count | 272 | 187 | 459 |
|  |  |  | Expected Count | 241.4 | 217.6 | 459.0 |
|  |  |  | Adjusted Residual | 5.1 | -5.1 |  |
|  | Total | | Count | 355 | 320 | 675 |
|  |  |  | Expected Count | 355.0 | 320.0 | 675.0 |
| M | Vm^2^ | L | Count | 21 | 8 | 29 |
|  |  |  | Expected Count | 14.2 | 14.8 | 29.0 |
|  |  |  | Adjusted Residual | 2.6 | -2.6 |  |
|  |  | M | Count | 47 | 41 | 88 |
|  |  |  | Expected Count | 43.2 | 44.8 | 88.0 |
|  |  |  | Adjusted Residual | .9 | -.9 |  |
|  |  | S | Count | 35 | 4 | 39 |
|  |  |  | Expected Count | 19.2 | 19.8 | 39.0 |
|  |  |  | Adjusted Residual | 5.2 | -5.2 |  |
|  |  | V | Count | 253 | 316 | 569 |
|  |  |  | Expected Count | 279.4 | 289.6 | 569.0 |
|  |  |  | Adjusted Residual | -4.8 | 4.8 |  |
|  | Total | | Count | 356 | 369 | 725 |
|  |  |  | Expected Count | 356.0 | 369.0 | 725.0 |
| Total | Vm^2^ | L | Count | 730 | 773 | 1503 |
|  |  |  | Expected Count | 741.9 | 761.1 | 1503.0 |
|  |  |  | Adjusted Residual | -.7 | .7 |  |
|  |  | M | Count | 340 | 346 | 686 |
|  |  |  | Expected Count | 338.6 | 347.4 | 686.0 |
|  |  |  | Adjusted Residual | ,1 | -,1 |  |
|  |  | S | Count | 572 | 623 | 1195 |
|  |  |  | Expected Count | 589.9 | 605.1 | 1195.0 |
|  |  |  | Adjusted Residual | -1.2 | 1.2 |  |
|  |  | V | Count | 675 | 635 | 1310 |
|  |  |  | Expected Count | 646.6 | 663.4 | 1310.0 |
|  |  |  | Adjusted Residual | 1.8 | -1.8 |  |
|  | Total | | Count | 2317 | 2377 | 4694 |
|  |  |  | Expected Count | 2317.0 | 2377.0 | 4694.0 |

Note. C = Cat; M = Mouse; P = Pitcher. Effort magnitude value (Vm^2^): Sedentary: S (0-2 CPS); Light L (<2-34CPS); Moderate M >34-100 CPS; Vigorous V (>100CPS).

| Chi-Square Tests | | | | |
| --- | --- | --- | --- | --- |
| ROLE | | value | df | Asymptotic Significance (2-sided) |
| C | Pearson Chi-Square | 38,199b | 3 | *p*< .001 |
|  | Likelihood Ratio | 39,844 | 3 | *p*< .001 |
|  | N of Valid Cases | 675 |  |  |
| M | Pearson Chi-Square | 37,632c | 3 | *p*< .001 |
|  | Likelihood Ratio | 41,479 | 3 | *p*< .001 |
|  | N of Valid Cases | 725 |  |  |
| P | Pearson Chi-Square | 2,825d | 3 | .419 |
|  | Likelihood Ratio | 2,825 | 3 | .419 |
|  | N of Valid Cases | 3294 |  |  |
| Total | Pearson Chi-Square | 3,914a | 3 | .271 |
|  | Likelihood Ratio | 3,915 | 3 | .271 |
|  | N of Valid Cases | 4694 |  |  |

**Table A11. Comparison of cyclic step time in V1 and V2**

|  | | | Pitcher_Version_1_2 | | Total |
| --- | --- | --- | --- | --- | --- |
|  |  |  | 1 | 2 |  |
| range_steps | HS | Count | 3 | 2 | 5 |
|  |  | % of Total | 0.1% | 0.0% | 0.1% |
|  |  | Adjusted Residual | ,5 | -,5 |  |
|  | LS | Count | 1880 | 1967 | 3847 |
|  |  | % of Total | 40.1% | 41.9% | 82.0% |
|  |  | Adjusted Residual | -1.4 | 1.4 |  |
|  | MS | Count | 116 | 103 | 219 |
|  |  | % of Total | 2.5% | 2.2% | 4.7% |
|  |  | Adjusted Residual | 1.1 | -1.1 |  |
|  | SS | Count | 318 | 305 | 623 |
|  |  | % of Total | 6.8% | 6.5% | 13.3% |
|  |  | Adjusted Residual | .9 | -.9 |  |
| Total | | Count | 2317 | 2377 | 4694 |
|  |  | % of Total | 49.4% | 50.6% | 100.0% |

Note. Steps: Low LS (0-1); Slight SS (2); Moderate MS (3) High HS (4).

| Chi-Square Tests | | | |
| --- | --- | --- | --- |
|  | value | df | Asymptotic Significance (2-sided) |
| Pearson Chi-Square | 2,444a | 3 | .486 |
| Likelihood Ratio | 2,446 | 3 | .485 |
| N of Valid Cases | 4694 |  |  |

Note. to. 2 cells (25.0%) have expected count less than 5. The minimum expected count is 2.47.

| Symmetric Measurements | | | |
| --- | --- | --- | --- |
|  | | value | Approximate Significance |
| Nominal by Nominal | Phi | .023 | .486 |
|  | Cramer's V | .023 | .486 |
| N of Valid Cases | | 4694 |  |

| **Table A12.** **Comparison of T-Patterns complexity in the V1 and V2 of the Elbow Tag game.** | | | | | | |
| --- | --- | --- | --- | --- | --- | --- |
| Complexity Subrole-Vm^2^-Steps-Relationships | | | | Pitcher_V1 V2 | | Total |
|  |  |  |  | V1 | V2 |  |
| 1 | T_Patterns | cc_v_ls_lr | Count | 1 | 0 | 1 |
|  |  |  | Adjusted Residual | 1,2 | -1,2 |  |
|  |  | ct_l_ls_lr | Count | 0 | 12 | 12 |
|  |  |  | Adjusted Residual | -2,9 | 2,9 |  |
|  |  | ct_m_ls_lr | Count | 0 | 4 | 4 |
|  |  |  | Adjusted Residual | -1,6 | 1,6 |  |
|  |  | ct_v_ls_lr | Count | 4 | 12 | 16 |
|  |  |  | Adjusted Residual | -1,3 | 1,3 |  |
|  |  | ct_v_ss_lr | Count | 6 | 15 | 21 |
|  |  |  | Adjusted Residual | -1,1 | 1,1 |  |
|  |  | ct_v_ss_mr | Count | 1 | 0 | 1 |
|  |  |  | Adjusted Residual | 1,2 | -1,2 |  |
|  |  | ct_v_ss_sr | Count | 1 | 0 | 1 |
|  |  |  | Adjusted Residual | 1,2 | -1,2 |  |
|  |  | mc_v_ss_lr | Count | 0 | 2 | 2 |
|  |  |  | Adjusted Residual | -1,2 | 1,2 |  |
|  |  | mj_v_ls_lr | Count | 9 | 0 | 9 |
|  |  |  | Adjusted Residual | 3,8 | -3,8 |  |
|  |  | mj_v_ss_lr | Count | 0 | 2 | 2 |
|  |  |  | Adjusted Residual | -1,2 | 1,2 |  |
|  |  | mx_l_ls_lr | Count | 3 | 0 | 3 |
|  |  |  | Adjusted Residual | 2,1 | -2,1 |  |
|  |  | mx_m_ls_lr | Count | 5 | 3 | 8 |
|  |  |  | Adjusted Residual | 1,3 | -1,3 |  |
|  |  | mx_s_ls_lr | Count | 5 | 0 | 5 |
|  |  |  | Adjusted Residual | 2,8 | -2,8 |  |
|  |  | mx_v_ls_lr | Count | 5 | 17 | 22 |
|  |  |  | Adjusted Residual | -1,7 | 1,7 |  |
|  |  | mx_v_ms_lr | Count | 2 | 2 | 4 |
|  |  |  | Adjusted Residual | ,4 | -,4 |  |
|  |  | mx_v_ss_lr | Count | 17 | 13 | 30 |
|  |  |  | Adjusted Residual | 2,0 | -2,0 |  |
|  |  | pl_l_ls_lr | Count | 3 | 4 | 7 |
|  |  |  | Adjusted Residual | ,2 | -,2 |  |
|  |  | pl_s_ls_lr | Count | 24 | 44 | 68 |
|  |  |  | Adjusted Residual | -,9 | ,9 |  |
|  | Total | | Count | 86 | 130 | 216 |

| 2 | T_Patterns | cc_v_ls_lr | Count | 3 | 0 | 3 |
| --- | --- | --- | --- | --- | --- | --- |
|  |  |  | Adjusted Residual | 2,1 | -2,1 |  |
|  |  | ct_l_ls_lr | Count | 0 | 6 | 6 |
|  |  |  | Adjusted Residual | -2,0 | 2,0 |  |
|  |  | ct_m_ls_lr | Count | 0 | 6 | 6 |
|  |  |  | Adjusted Residual | -2,0 | 2,0 |  |
|  |  | ct_s_ls_lr | Count | 0 | 2 | 2 |
|  |  |  | Adjusted Residual | -1,2 | 1,2 |  |
|  |  | ct_v_ls_lr | Count | 7 | 10 | 17 |
|  |  |  | Adjusted Residual | ,1 | -,1 |  |
|  |  | ct_v_ls_mr | Count | 1 | 0 | 1 |
|  |  |  | Adjusted Residual | 1,2 | -1,2 |  |
|  |  | ct_v_ms_lr | Count | 0 | 1 | 1 |
|  |  |  | Adjusted Residual | -,8 | ,8 |  |
|  |  | ct_v_ss_lr | Count | 8 | 21 | 29 |
|  |  |  | Adjusted Residual | -1,4 | 1,4 |  |
|  |  | ct_v_ss_mr | Count | 1 | 0 | 1 |
|  |  |  | Adjusted Residual | 1,2 | -1,2 |  |
|  |  | mc_v_ls_lr | Count | 3 | 1 | 4 |
|  |  |  | Adjusted Residual | 1,5 | -1,5 |  |
|  |  | mc_v_ms_lr | Count | 3 | 1 | 4 |
|  |  |  | Adjusted Residual | 1,5 | -1,5 |  |
|  |  | mc_v_ss_lr | Count | 7 | 16 | 23 |
|  |  |  | Adjusted Residual | -1,0 | 1,0 |  |
|  |  | mj_v_ls_lr | Count | 4 | 0 | 4 |
|  |  |  | Adjusted Residual | 2,5 | -2,5 |  |
|  |  | mx_m_ls_lr | Count | 5 | 3 | 8 |
|  |  |  | Adjusted Residual | 1,3 | -1,3 |  |
|  |  | mx_s_ls_lr | Count | 2 | 0 | 2 |
|  |  |  | Adjusted Residual | 1,7 | -1,7 |  |
|  |  | mx_v_ls_lr | Count | 13 | 34 | 47 |
|  |  |  | Adjusted Residual | -1,9 | 1,9 |  |
|  |  | mx_v_ms_lr | Count | 8 | 7 | 15 |
|  |  |  | Adjusted Residual | 1,1 | -1,1 |  |
|  |  | mx_v_ss_lr | Count | 21 | 18 | 39 |
|  |  |  | Adjusted Residual | 2,0 | -2,0 |  |
|  |  | pl_s_ls_lr | Count | 0 | 2 | 2 |
|  |  |  | Adjusted Residual | -1,2 | 1,2 |  |
|  | Total | | Count | 86 | 130 | 216 |
| 3 | T_Patterns | cc_v_ls_lr | Count | 2 | 0 | 2 |
|  |  |  | Adjusted Residual | 2,1 | -2,1 |  |
|  |  | ct_v_ls_lr | Count | 1 | 2 | 3 |
|  |  |  | Adjusted Residual | ,0 | ,0 |  |
|  |  | ct_v_ls_mr | Count | 1 | 0 | 1 |
|  |  |  | Adjusted Residual | 1,5 | -1,5 |  |
|  |  | ct_v_ms_lr | Count | 0 | 1 | 1 |
|  |  |  | Adjusted Residual | -,7 | ,7 |  |
|  |  | ct_v_ss_lr | Count | 4 | 3 | 7 |
|  |  |  | Adjusted Residual | 1,5 | -1,5 |  |
|  |  | mc_v_ms_lr | Count | 2 | 0 | 2 |
|  |  |  | Adjusted Residual | 2,1 | -2,1 |  |
|  |  | mc_v_ss_lr | Count | 3 | 19 | 22 |
|  |  |  | Adjusted Residual | -2,1 | 2,1 |  |
|  |  | mx_m_ls_lr | Count | 1 | 0 | 1 |
|  |  |  | Adjusted Residual | 1,5 | -1,5 |  |
|  |  | mx_v_ls_lr | Count | 5 | 17 | 22 |
|  |  |  | Adjusted Residual | -1,1 | 1,1 |  |
|  |  | mx_v_ms_lr | Count | 0 | 3 | 3 |
|  |  |  | Adjusted Residual | -1,2 | 1,2 |  |
|  |  | mx_v_ss_lr | Count | 8 | 16 | 24 |
|  |  |  | Adjusted Residual | ,1 | -,1 |  |
|  |  | pl_l_ls_lr | Count | 1 | 0 | 1 |
|  |  |  | Adjusted Residual | 1,5 | -1,5 |  |
|  |  | pl_s_ls_lr | Count | 3 | 4 | 7 |
|  |  |  | Adjusted Residual | ,6 | -,6 |  |
|  | Total | | Count | 31 | 65 | 96 |
| 4 | T_Patterns | ct_l_ls_lr | Count | 0 | 2 | 2 |
|  |  |  | Adjusted Residual | -,9 | ,9 |  |
|  |  | ct_v_ls_lr | Count | 0 | 2 | 2 |
|  |  |  | Adjusted Residual | -,9 | ,9 |  |
|  |  | ct_v_ss_lr | Count | 0 | 1 | 1 |
|  |  |  | Adjusted Residual | -,6 | ,6 |  |
|  |  | mc_v_ss_lr | Count | 0 | 2 | 2 |
|  |  |  | Adjusted Residual | -,9 | ,9 |  |
|  |  | mx_m_ls_lr | Count | 2 | 0 | 2 |
|  |  |  | Adjusted Residual | 2,4 | -2,4 |  |
|  |  | mx_v_ls_lr | Count | 1 | 1 | 2 |
|  |  |  | Adjusted Residual | ,7 | -,7 |  |
|  |  | mx_v_ms_lr | Count | 0 | 1 | 1 |
|  |  |  | Adjusted Residual | -,6 | ,6 |  |
|  |  | mx_v_ss_lr | Count | 3 | 3 | 6 |
|  |  |  | Adjusted Residual | 1,4 | -1,4 |  |
|  |  | pl_s_ls_lr | Count | 0 | 3 | 3 |
|  |  |  | Adjusted Residual | -1,2 | 1,2 |  |
|  | Total | | Count | 6 | 15 | 21 |
| 5 | T_Patterns | ct_l_ls_lr | Count |  | 1 | 1 |
|  |  |  | Adjusted Residual |  | . |  |
|  |  | mc_v_ss_lr | Count |  | 1 | 1 |
|  |  |  | Adjusted Residual |  | . |  |
|  |  | mx_v_ls_lr | Count |  | 4 | 4 |
|  |  |  | Adjusted Residual |  | . |  |
|  |  | mx_v_ss_lr | Count |  | 1 | 1 |
|  |  |  | Adjusted Residual |  | . |  |
|  |  | pl_s_ls_lr | Count |  | 1 | 1 |
|  |  |  | Adjusted Residual |  | . |  |
|  | Total | | Count |  | 8 | 8 |
| 6 | T_Patterns | mc_v_ss_lr | Count |  | 1 | 1 |
|  |  |  | Adjusted Residual |  | . |  |
|  |  | mx_v_ls_lr | Count |  | 1 | 1 |
|  |  |  | Adjusted Residual |  | . |  |
|  | Total | | Count |  | 2 | 2 |
| Total | T_Patterns | cc_v_ls_lr | Count | 6 | 0 | 6 |
|  |  |  | Adjusted Residual | 3,2 | -3,2 |  |
|  |  | ct_l_ls_lr | Count | 0 | 21 | 21 |
|  |  |  | Adjusted Residual | -3,6 | 3,6 |  |
|  |  | ct_m_ls_lr | Count | 0 | 10 | 10 |
|  |  |  | Adjusted Residual | -2,5 | 2,5 |  |
|  |  | ct_s_ls_lr | Count | 0 | 2 | 2 |
|  |  |  | Adjusted Residual | -1,1 | 1,1 |  |
|  |  | ct_v_ls_lr | Count | 12 | 26 | 38 |
|  |  |  | Adjusted Residual | -,8 | ,8 |  |
|  |  | ct_v_ls_mr | Count | 2 | 0 | 2 |
|  |  |  | Adjusted Residual | 1,8 | -1,8 |  |
|  |  | ct_v_ms_lr | Count | 0 | 2 | 2 |
|  |  |  | Adjusted Residual | -1,1 | 1,1 |  |
|  |  | ct_v_ss_lr | Count | 18 | 40 | 58 |
|  |  |  | Adjusted Residual | -1,1 | 1,1 |  |
|  |  | ct_v_ss_mr | Count | 2 | 0 | 2 |
|  |  |  | Adjusted Residual | 1,8 | -1,8 |  |
|  |  | ct_v_ss_sr | Count | 1 | 0 | 1 |
|  |  |  | Adjusted Residual | 1,3 | -1,3 |  |
|  |  | mc_v_ls_lr | Count | 3 | 1 | 4 |
|  |  |  | Adjusted Residual | 1,6 | -1,6 |  |
|  |  | mc_v_ms_lr | Count | 5 | 1 | 6 |
|  |  |  | Adjusted Residual | 2,3 | -2,3 |  |
|  |  | mc_v_ss_lr | Count | 10 | 41 | 51 |
|  |  |  | Adjusted Residual | -2,8 | 2,8 |  |
|  |  | mj_v_ls_lr | Count | 13 | 0 | 13 |
|  |  |  | Adjusted Residual | 4,7 | -4,7 |  |
|  |  | mj_v_ss_lr | Count | 0 | 2 | 2 |
|  |  |  | Adjusted Residual | -1,1 | 1,1 |  |
|  |  | mr_v_ls_lr | Count | 0 | 2 | 2 |
|  |  |  | Adjusted Residual | -1,1 | 1,1 |  |
|  |  | mx_l_ls_lr | Count | 3 | 0 | 3 |
|  |  |  | Adjusted Residual | 2,2 | -2,2 |  |
|  |  | mx_m_ls_lr | Count | 13 | 6 | 19 |
|  |  |  | Adjusted Residual | 2,8 | -2,8 |  |
|  |  | mx_s_ls_lr | Count | 7 | 0 | 7 |
|  |  |  | Adjusted Residual | 3,4 | -3,4 |  |
|  |  | mx_v_ls_lr | Count | 24 | 74 | 98 |
|  |  |  | Adjusted Residual | -2,9 | 2,9 |  |
|  |  | mx_v_ms_lr | Count | 10 | 13 | 23 |
|  |  |  | Adjusted Residual | ,6 | -,6 |  |
|  |  | mx_v_ss_lr | Count | 49 | 51 | 100 |
|  |  |  | Adjusted Residual | 2,6 | -2,6 |  |
|  |  | pl_l_ls_lr | Count | 4 | 4 | 8 |
|  |  |  | Adjusted Residual | ,7 | -,7 |  |
|  |  | pl_s_ls_lr | Count | 27 | 54 | 81 |
|  |  |  | Adjusted Residual | -,8 | ,8 |  |
|  | Total | | Count | 209 | 350 | 559 |

| Case Processing Summary | | | | | | |
| --- | --- | --- | --- | --- | --- | --- |
|  | Cases | | | | | |
|  | Valid | | Missing | | Total | |
|  | N | Percent | N | Percent | N | Percent |
| T_Patterns * Elbow Tag V1 V2 * Complexity | 559 | 100,0% | 0 | 0,0% | 559 | 100,0% |

| Chi-Square Tests | | | | |
| --- | --- | --- | --- | --- |
| Complexity | | Value | df | Asymptotic Significance (2-sided) |
| 1 | Pearson Chi-Square | 54,771^b^ | 17 | *p*< .001 |
|  | Likelihood Ratio | 68,667 | 17 | *p*< .001 |
|  | N of Valid Cases | 216 |  |  |
| 2 | Pearson Chi-Square | 44,962^c^ | 19 | *p*< .001 |
|  | Likelihood Ratio | 55,372 | 19 | *p*< .001 |
|  | N of Valid Cases | 216 |  |  |
| 3 | Pearson Chi-Square | 23,355^d^ | 12 | ,025 |
|  | Likelihood Ratio | 26,176 | 12 | ,010 |
|  | N of Valid Cases | 96 |  |  |
| 4 | Pearson Chi-Square | 11,200^e^ | 8 | ,191 |
|  | Likelihood Ratio | 14,037 | 8 | ,081 |
|  | N of Valid Cases | 21 |  |  |
| 5 | Pearson Chi-Square | .^f^ |  |  |
|  | N of Valid Cases | 8 |  |  |
| 6 | Pearson Chi-Square | .^f^ |  |  |
|  | N of Valid Cases | 2 |  |  |
| Total | Pearson Chi-Square | 118,507^a^ | 23 | *p*< .001 |
|  | Likelihood Ratio | 142,262 | 23 | *p*< .001 |
|  | N of Valid Cases | 559 |  |  |

| Symmetric Measures | | | | |
| --- | --- | --- | --- | --- |
| Complexity | | | Value | Approximate Significance |
| 1 | Nominal by Nominal | Phi | ,504 | *p*< .001 |
|  |  | Cramer's V | ,504 | *p*< .001 |
|  | N of Valid Cases | | 216 |  |
| 2 | Nominal by Nominal | Phi | ,456 | *p*< .001 |
|  |  | Cramer's V | ,456 | *p*< .001 |
|  | N of Valid Cases | | 216 |  |
| 3 | Nominal by Nominal | Phi | ,493 | ,025 |
|  |  | Cramer's V | ,493 | ,025 |
|  | N of Valid Cases | | 96 |  |
| 4 | Nominal by Nominal | Phi | ,730 | ,191 |
|  |  | Cramer's V | ,730 | ,191 |
|  | N of Valid Cases | | 21 |  |
| 5 | Nominal by Nominal | Phi | .^c^ |  |
|  | N of Valid Cases | | 8 |  |
| 6 | Nominal by Nominal | Phi | .^c^ |  |
|  | N of Valid Cases | | 2 |  |
| Total | Nominal by Nominal | Phi | ,460 | *p*< .001 |
|  |  | Cramer's V | ,460 | *p*< .001 |
|  | N of Valid Cases | | 559 |  |

**Table A13.** **Cross Table. Temporary strategies of the Mouse**

|  | | | VersionNum | | Total |
| --- | --- | --- | --- | --- | --- |
|  |  |  | 1 | 2 |  |
| C_ET_R1 | 2 | Count | 58 | 68 | 126 |
|  |  | Adjusted Residual | -1.1 | 1.1 |  |
|  | 3 | Count | 31 | 29 | 60 |
|  |  | Adjusted Residual | ,4 | -,4 |  |
|  | 4 | Count | 40 | 35 | 75 |
|  |  | Adjusted Residual | .8 | -.8 |  |
| Total | | Count | 129 | 132 | 261 |

Note. 2. Go quickly to the chosen Pitcher; 3 Take some time before going to the chosen Pitcher. Play with the cat; 4 Both strategies depending on the play.

| Chi-Square Tests | | | |
| --- | --- | --- | --- |
|  | value | df | Asymptotic Significance (2-sided) |
| Pearson Chi-Square | 1,159a | 2 | ,560 |
| Likelihood Ratio | 1,160 | 2 | ,560 |
| Linear-by-Linear Association | 1,084 | 1 | .298 |
| N of Valid Cases | 261 |  |  |

Note. to. 0 cells (0.0%) have expected count less than 5. The minimum expected count is 29.66.

| Symmetric Measurements | | | |
| --- | --- | --- | --- |
|  | | value | Approximate Significance |
| Nominal by Nominal | Phi | .067 | ,560 |
|  | Cramer's V | .067 | ,560 |
| N of Valid Cases | | 261 |  |

**Table A14.** **Intensity in the five basic emotions in the three for V1 and V2.**

| Descriptives | | | | | | | |
| --- | --- | --- | --- | --- | --- | --- | --- |
|  | | **Version** | | **mean** | | **S.D.** | |
| Cat_Joy |  | V1 |  | 4.44 |  | 1.800 |  |
|  |  | V2 |  | 4.87 |  | 1.644 |  |
| Cat_Anger |  | V1 |  | 2.23 |  | 1.584 |  |
|  |  | V2 |  | 2.06 |  | 1.433 |  |
| Cat_Sadness |  | V1 |  | 1.35 |  | 0.889 |  |
|  |  | V2 |  | 1.21 |  | 0.640 |  |
| Cat_Fear |  | V1 |  | 1.31 |  | 0.944 |  |
|  |  | V2 |  | 1.49 |  | 1.178 |  |
| Cat_Rejection |  | V1 |  | 1.39 |  | 0.971 |  |
|  |  | V2 |  | 1.30 |  | 0.903 |  |
| Cat_ Negative |  | V1 |  | 1.57 |  | 0.796 |  |
|  |  | V2 |  | 1.51 |  | 0.774 |  |
| Mouse_Joy |  | V1 |  | 5.31 |  | 1.631 |  |
|  |  | V2 |  | 5.23 |  | 1.611 |  |
| Mouse_ Anger |  | V1 |  | 1.54 |  | 0.985 |  |
|  |  | V2 |  | 1.68 |  | 1.177 |  |
| Mouse_Sadness |  | V1 |  | 1.18 |  | 0.540 |  |
|  |  | V2 |  | 1.16 |  | 0.583 |  |
| Mouse_Fear |  | V1 |  | 1.86 |  | 1.400 |  |
|  |  | V2 |  | 1.74 |  | 1.279 |  |
| Mouse_Rejection |  | V1 |  | 1.18 |  | 0.579 |  |
|  |  | V2 |  | 1.13 |  | 0.413 |  |
| Mouse_ Negative |  | V1 |  | 1.44 |  | 0.577 |  |
|  |  | V2 |  | 1.43 |  | 0.557 |  |
| Pitcher_Joy |  | V1 |  | 4.59 |  | 1.823 |  |
|  |  | V2 |  | 4.46 |  | 1.832 |  |
| Pitcher_ Anger |  | V1 |  | 1.65 |  | 1.297 |  |
|  |  | V2 |  | 1.71 |  | 1.311 |  |
| Pitcher_Sadness |  | V1 |  | 1.34 |  | 0.888 |  |
|  |  | V2 |  | 1.29 |  | 0.682 |  |
| Pitcher_Fear |  | V1 |  | 1.71 |  | 1.225 |  |
|  |  | V2 |  | 1.53 |  | 1.109 |  |
| Pitcher_Rejection |  | V1 |  | 1.46 |  | 1.108 |  |
|  |  | V2 |  | 1.34 |  | 0.880 |  |
| Pitcher_Negative |  | V1 |  | 1.54 |  | 0.687 |  |
|  |  | V2 |  | 1.47 |  | 0.682 |  |
